# A high-energy X-ray beamline for materials science research at SPring-8: BL15XU

**DOI:** 10.1107/S1600577526006387

**Published:** 2026-07-27

**Authors:** Hiroyuki Ohsumi, Yuji Higo, Yujiro Hayashi, Jaemyung Kim, Noriyoshi Tsujino, Sho Kakizawa, Tetsuo Irifune, Tomohiro Ohuchi, Yoshio Kono, Hirokatsu Yumoto, Takahisa Koyama, Hiroshi Yamazaki, Yasunori Senba, Haruhiko Ohashi, Hidekazu Takano, Michihiro Sugahara, Taito Osaka, Makina Yabashi

**Affiliations:** a RIKEN SPring-8 Center, 1-1-1 Kouto, Sayo-cho, Sayo-gun, Hyogo679-5148, Japan; b Japan Synchrotron Radiation Research Institute (JASRI) 1-1-1 Kouto Sayo-cho, Sayo-gun Hyogo679-5198 Japan; c Geodynamics Research Center Ehime University Matsuyama Japan; d Earth-Life Science Institute Tokyo Institute of Technology Tokyo Japan; e Department of Physics and Astronomy Kwansei Gakuin University Sanda Japan; Brazilian Synchrotron Light Laboratory, Brazil

**Keywords:** lamino­graphy, scanning 3DXRD, large-volume press, deformation presses, pair distribution function analysis

## Abstract

The high-energy X-ray beamline BL15XU is designed to drive advancements in materials science and high-pressure research by leveraging the photon source characteristics of SPring-8. The beamline provides an intense 100 keV pink beam, extracted from undulator radiation using a double-multilayer monochromator, and delivers a high flux of 6.0 × 10^13^ photons s^−1^ to the sample position. Experimental hutch 1 is dedicated to materials engineering, employing non-destructive methods based on scanning 3D X-ray diffraction and computed lamino­graphic imaging. Experimental hutch 2 focuses on high-pressure science, and is equipped with a rotational slit system capable of switching between radiography and diffraction modes at speeds up to 144 Hz, with a minimum exposure time of approximately 2.7 ms. Installed high-pressure apparatuses include: the 1500 ton MADONNA multi-anvil press for generating pressures up to 120 GPa, the mobile 200 ton Hyaku-shiki press for *in situ* deformation experiments up to 20 GPa, and a dedicated Paris–Edinburgh press setup for pair distribution function analysis of liquids and amorphous materials over a wide momentum transfer range up to 27.8 Å^−1^. These diverse capabilities establish BL15XU as a versatile platform for *in situ* investigations of materials science and high-pressure research, well prepared for future high-brilliance operations at SPring-8-II.

## Abstract 2

The Japanese synchrotron facility SPring-8 has recently relaunched BL15XU as a high-energy X-ray beamline for materials science research. A comprehensive description of the technical details and science cases is presented.

## 1. Introduction

Increasing X-ray photon energy improves both penetration depth and accessible momentum transfer. These features play a key role in certain materials science research techniques, such as radiography and total scattering. SPring-8 holds a distinct advantage in generating high-energy X-rays because it has the world’s highest electron acceleration energy as a synchrotron light source. As electrons approach the speed of light, the wavelength of undulator radiation is compressed further by the Doppler shift; also, its brightness increases due to relativistic beaming. To fully leverage this potential, sophisticated high-energy X-ray optics and utilization techniques were developed at BL05XU (Yumoto *et al.*, 2025 ▸). These advancements have been integrated into a new dedicated beamline.

BL15XU is a beamline designed to promote materials science research that requires ultra-brilliant, high-energy X-rays. An intense 100 keV pink beam is provided by extracting a single harmonic radiation from an in-vacuum undulator (IVU) using a double-multilayer monochromator (DMM). Employing a DMM instead of a double-crystal monochromator with Si perfect crystals significantly increases photon flux by more than two orders of magnitude. The photon energy of 100 keV is exceptionally high compared with other insertion device beamlines where one can use a pink beam (Wilde *et al.*, 2016 ▸; Weitkamp *et al.*, 2017 ▸; Rau *et al.*, 2019 ▸; Mezouar *et al.*, 2024 ▸). The beamline assembles various apparatuses for photon-hungry experiments in materials engineering and high-pressure science. Here we present a new configuration of the beamline, which underwent reconstruction following the termination of the NIMS contract beamline (Ueda *et al.*, 2010 ▸). To ensure a seamless restart following the upgrade to SPring-8-II (Tanaka *et al.*, 2024 ▸), the beamline has been outfitted with a photon source and beamline optics tailored for the new accelerator. Experimental hutches have been rebuilt to meet the radiation-shielding requirements for a 100 keV pink beam.

Materials engineering research is one primary focus area addressed by the beamline. Experimental hutch 1 (EH1) accommodates specialized apparatuses designed for scanning 3D X-ray diffraction and computed lamino­graphic imaging. The former is a powerful technique for investigating polycrystalline grains in terms of their position, orientation, shape and stress, essential information for analysing fatigue in polycrystalline alloys ubiquitous in industrial products. The apparatus is equipped with a scanning mechanism that adjusts the position of a bulk specimen relative to a confocal gauge volume. A 100 keV pink beam facilitates non-destructive inspection of larger specimens, such as 10 mm-thick steel, surpassing the size limitations currently in place (Hayashi *et al.*, 2019 ▸; Hektor *et al.*, 2019 ▸; Henningsson *et al.*, 2024 ▸). Computed lamino­graphic imaging, a non-destructive observation technique similar to computed tomography, is best suited for analysing failures of electrical parts or power devices on large, flat circuit boards.

Experimental hutch 2 (EH2) focuses on high-pressure science research, equipped with a 1500 ton large-volume press that facilitates investigation into mantle minerals under pressure conditions equivalent to those of the deep lower mantle of Earth. Pseudo-concurrent data acquisition between radiography and diffractometry can be achieved by using spatiotemporally structured X-rays and alternate gating techniques. A rotational slit system allows for adjusting aperture size at desired frequencies. For applications where the large press is not suitable, such as acoustic emission (AE) monitoring, a 200 ton large-volume press with a high-speed and low-noise stage system is also available. In addition, a Paris–Edinburgh press with a dedicated diffractometer offers high-*Q*-range measurements of the structure factor of liquids and glasses up to 27.8 Å^−1^. Compared with other major synchrotron facilities such as ESRF, APS and PETRA III, BL15XU leverages a 100 keV pink beam, offering a distinct advantage in high-speed time-resolved measurements due to its high photon flux. This versatile platform supports both deep-Earth geoscience, such as dynamic mineral deformation, and materials science, focusing on high-pressure synthesis of novel materials and structural characterization over a wider *Q* range.

## 2. Beamline overview

### 2.1. Photon source

A photon source of the beamline is the in-vacuum undulator for SPring-8-II (Imamura *et al.*, 2024 ▸) with a magnetic period of 22 mm (IVU-II22). The device has 149 periods, resulting in a magnetic length of 3.278 m, which fits within the shorter straight section of SPring-8-II. At the current SPring-8 facility, the IVU-II22 yields a brilliance of 2 × 10^19^ photons s^−1^ mm^−2^ mrad^−2^ (0.1% bandwidth)^−1^ at 100 keV. A maximum flux is achieved by the seventh harmonic of undulator radiation with a magnetic gap of 8.744 mm (*K* = 1.36). Upon upgrading to SPring-8-II, emittance of the electron beam will be significantly reduced using multi-bend achromat technologies, leading to an improved brilliance at 100 keV of up to 2×10^20^ photons s^−1^ mm^−2^ mrad^−2^ (0.1%bandwidth)^−1^. The maximum flux will be attained through the 19th harmonic of undulator radiation with a magnetic gap of 6.104 mm (*K* = 1.97). Additionally, the significant improvement in horizontal electron beam emittance will reduce off-axis spectral components and their leakage onto the on-axis spectrum as a tail on the long-wavelength side. Such a well defined, separated single harmonic is ideal for a pink beam source. An energy bandwidth of each harmonic typically amounts to approximately 1%, although this can vary depending on various factors. Table 1 ▸ summarizes the main source parameters of IVU-II22 at SPring-8 and SPring-8-II.

### 2.2. Beamline optics

**Figure 1:**
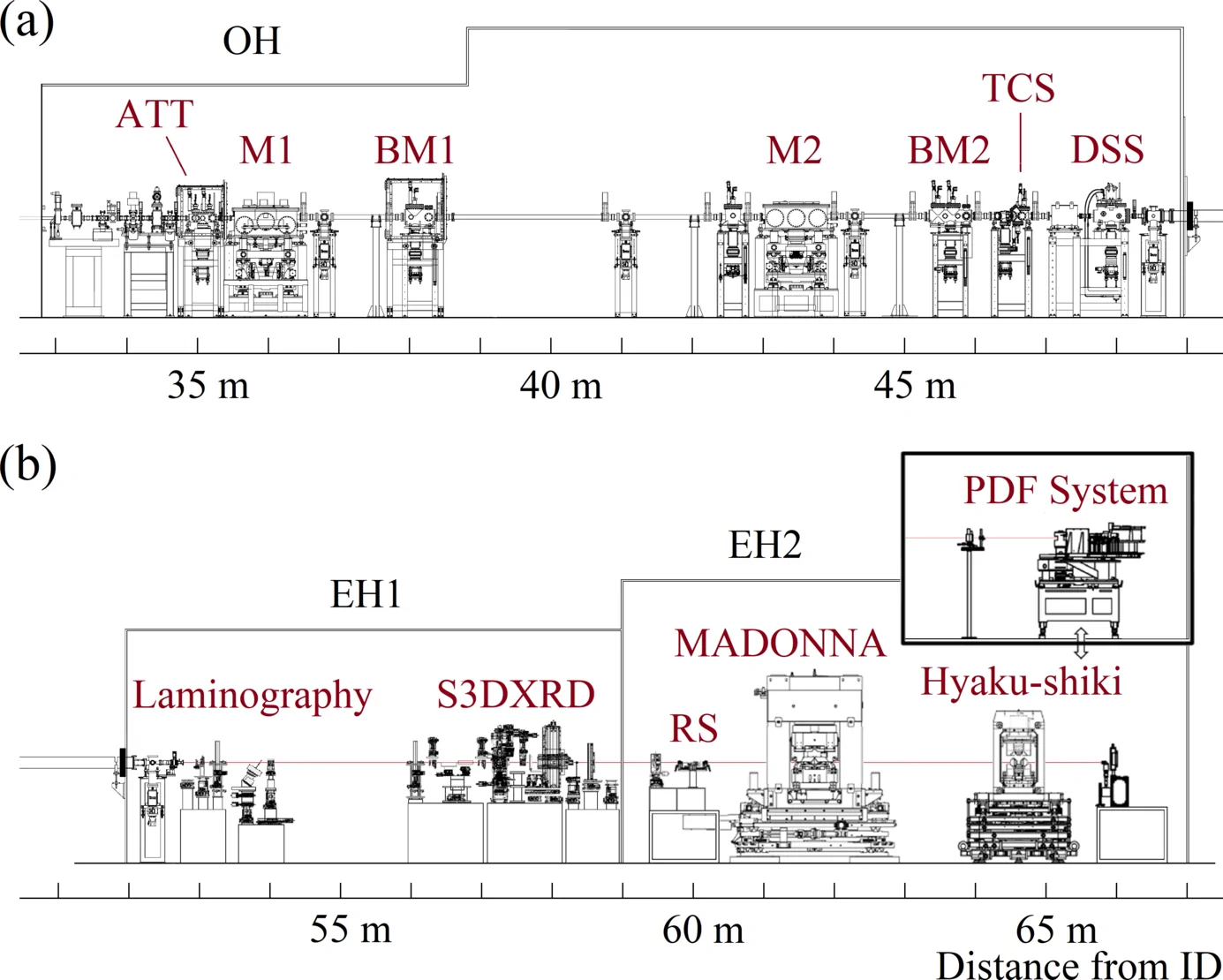
Layout of the reconstructed BL15XU at SPring-8. (*a*) Components in the optics hutch. ATT: attenuator; M (M1, M2): mirror; BM (BM1, BM2): beam profile monitor; TCS: transport channel slit; DSS: downstream shutter. (*b*) Apparatuses in experimental hutches. RS: rotational slit.

The beamline consists of one optics hutch (OH) and two experimental hutches. Fig. 1 ▸(*a*) depicts the schematic layout inside the OH. All X-ray optics of the beamline were replaced for producing a 100 keV pink beam. The first optical element following the front-end section is attenuator plates (ATTs), comprising diamond (1.2 mm), SiC (1.4 mm), Si (8.0 mm) and Mo (0.2 mm). The ATTs stack provides thermal protection for subsequent X-ray optics and eliminates low-energy photons subject to total reflection at the DMM. The high-pass-filtered undulator radiation is quasi-monochromated using the DMM into a 100 keV pink beam with a 1% bandwidth. The mirrors, consisting of 150 pairs of 3.17 nm periodic Cr/C multilayers, were newly fabricated based on those at BL05XU (Yumoto *et al.*, 2025 ▸). The multilayers cause first-order diffraction at an incident angle of 2.0 mrad, restricting the vertical beam acceptance to 1100 µm. Consequently, the front-end slit aperture is confined to 0.58 mm in height and 1.52 mm in width. To mitigate thermal deformation effects, the first mirror (M1) of the DMM is equipped with a liquid-nitro­gen cooling system. The simulated flux of 100 keV photons leaving the second mirror (M2) is 1 × 10^14^ photons s^−1^ at SPring-8 and 7 × 10^13^ photons s^−1^ at SPring-8-II, as calculated using the *SPECTRA* code (Tanaka, 2021 ▸). The measured flux at the sample position in EH1, passing through a transport channel slit (TCS), a downstream shutter (DSS) and exit windows, was 6.0 × 10^13^ photons s^−1^. Two beam profile monitors (BM1 and BM2) are installed for beam inspection. BM1 is situated between M1 and M2, and BM2 is positioned between M2 and DSS. These beam monitors have fluorescent screens made of single-crystalline diamond, which are temporarily inserted into the beam axis during inspection. Fluorescent images on the screens can be observed through viewports using a CMOS camera.

### 2.3. EH1 and apparatuses

Fig. 1 ▸(*b*) illustrates the layout of apparatuses inside EH1 and EH2. A monochromated beam from the DMM enters EH1 through a 100 µm-thick beryllium window and a 25 µm-thick graphite exit window. The later window functions as an airlock to place the beryllium window in a vacuum. The primary focus of EH1 lies in applications related to materials engineering. An apparatus for computed lamino­graphic imaging, which is also capable of operating in tomography geometry, is installed just downstream from the exit windows. The aim behind incorporating an incline mechanism into a rotation axis is to enable reliability evaluations of electronic component assemblies on large, flat circuit boards. A non-destructive 3D inspection of dense planar specimens, unsuitable for computed tomography, boasts a resolution of approximately 2 µm and a detector field of view of 712.4 µm × 630.2 µm. This industrial-oriented apparatus will be described in detail elsewhere. In addition to this, EH1 accommodates a scanning 3D X-ray diffraction (S3DXRD) measurement system. This large-scale system, installed on the downstream side, allows for comprehensive visualization of the 3D metallographic structure of polycrystalline materials. Fig. 1 ▸(*b*) shows an unoccupied space between the two apparatuses, providing room for future introduction of additional apparatus.

#### 2.3.1. S3DXRD measurement system

**Figure 2:**
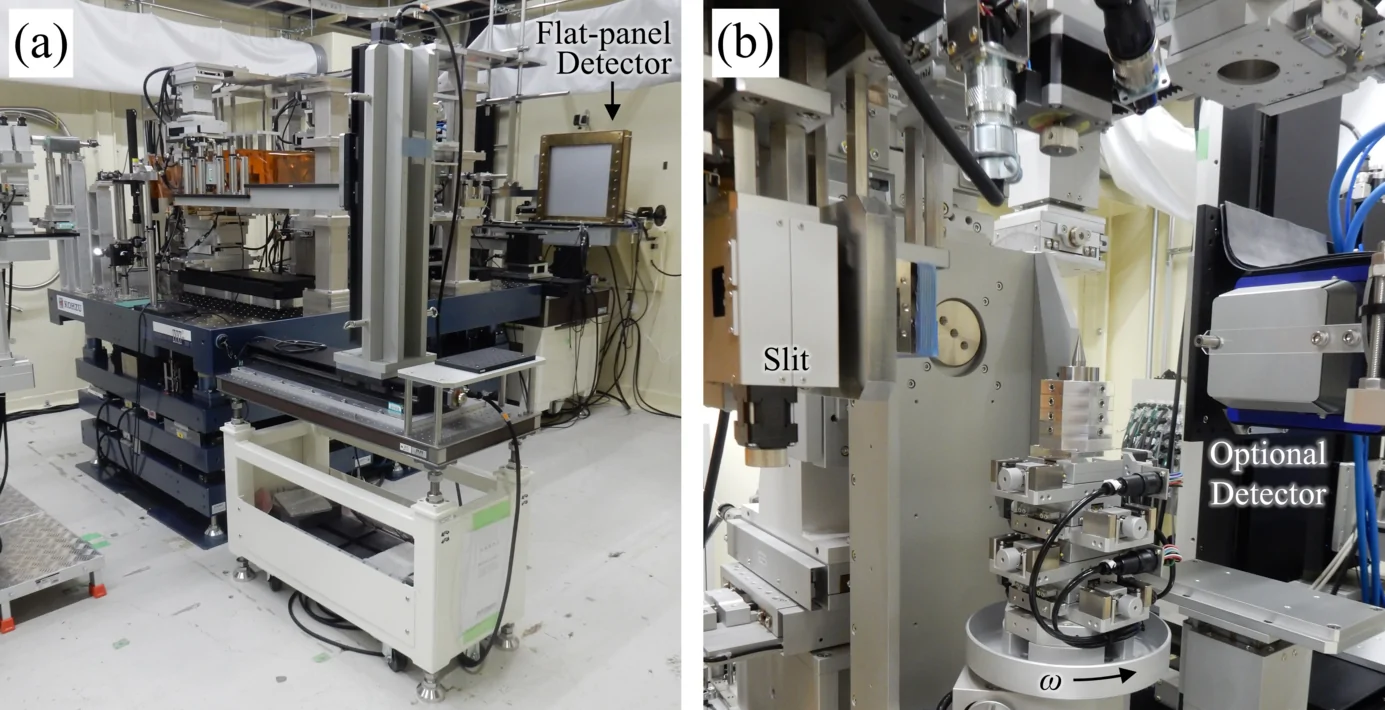
S3DXRD measurement system. (*a*) Overall view of the apparatus. (*b*) Incident four-quadrant slit and sample stages. An optional 2D detector enables high-throughput data acquisition, although the range of solid angle is limited.

S3DXRD (Hayashi *et al.*, 2015 ▸; Hayashi *et al.*, 2019 ▸; Hektor *et al.*, 2019 ▸; Henningsson *et al.*, 2020 ▸; Henningsson *et al.*, 2024 ▸; Kim *et al.*, 2023*a* ▸; Kim *et al.*, 2023*b* ▸) is one of the primary measurement techniques at EH1 for investigating industrial-relevant polycrystalline specimens. Fig. 2 ▸ shows photographs of the S3DXRD measurement system. A 100 keV pink beam from the DMM is shaped using a four-quadrant slit, located 230 mm upstream of a sample rotation centre. The sample is rotated using an ω stage, driven by a stepping motor and mounted on a motorized *X*–*Z* translation stage. For fine alignment of the sample, additional *x*_s_–*y*_s_ micro-positioning stages are placed above the ω stage. A flat-panel detector XRD4343CT (Varex Imaging) with a 150 µm pixel size was installed 1.45 m downstream of the sample rotation centre. Alternatively, a smaller-area high-speed 2D detector can be used for faster data acquisition, although it covers a smaller solid angle. Conical slits can be installed to measure relatively thick polycrystalline materials, such as steel bars, by reducing the gauge volume. Conversely, the absence of conical slits makes the setup more suitable for orientation mapping of thin specimens, including metal wires of small tensile-test pieces. In addition, a multilayer Kirkpatrick–Baez focusing system (Koyama *et al.*, 2024 ▸) is being relocated for sub-micrometre focusing.

**Figure 3:**
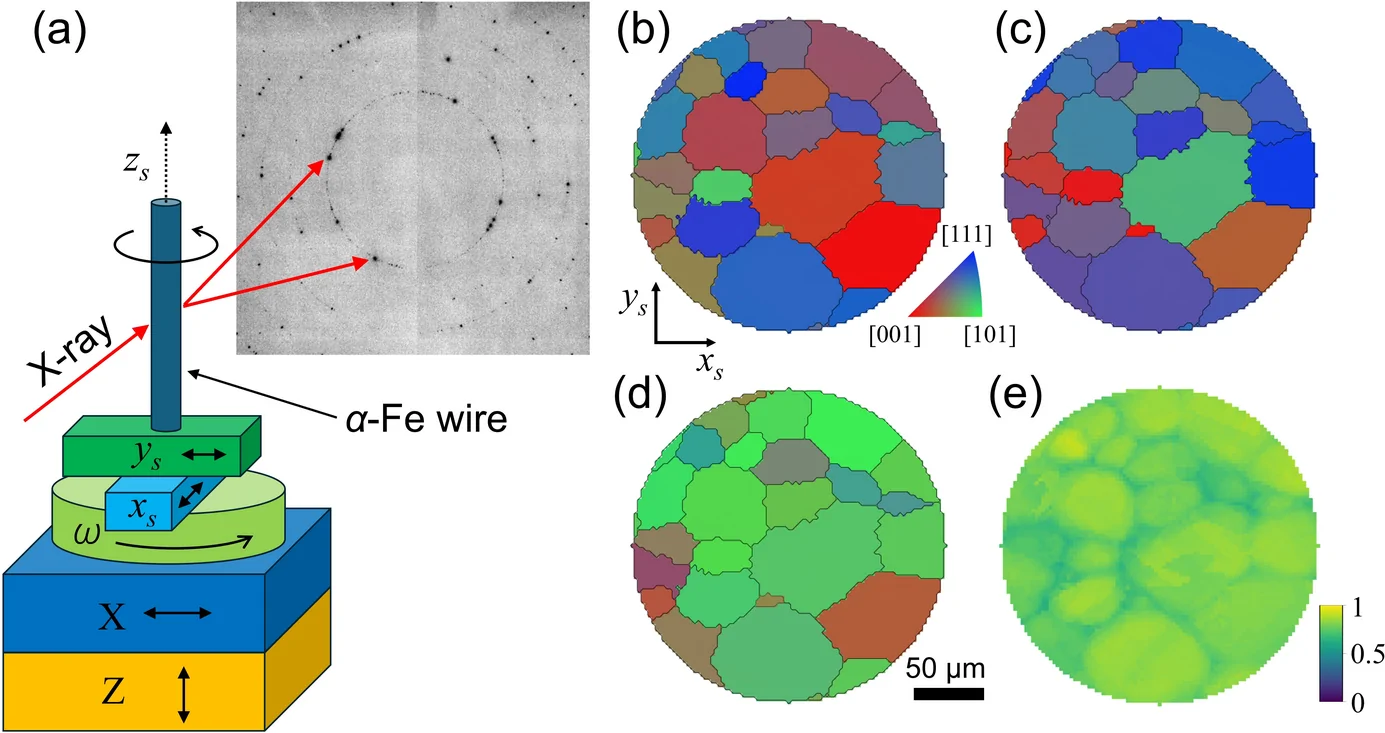
S3DXRD configuration and reconstructed maps of the α-Fe wire. (*a*) Experimental setup at EH1 for S3DXRD. A rotation stage (ω) is mounted on the *X*–*Z* translation stage, allowing the lateral shift of the rotation axis with the *X* stage. Above the rotation stage, a pair of *x*_s_–*y*_s_ alignment stages supports the positioning of the α-Fe specimen. (*b*) Reconstructed IPF-*x*_s_ and (*c*) IPF-*y*_s_ maps show a seemingly random arrangement of grain orientations, whereas (*d*) IPF-*z*_s_ map displays a grain texture with a preferred orientation. (*e*) Completeness map clearly highlights the grain boundaries in the polycrystalline α-Fe wire.

To demonstrate the system’s capabilities, we conducted S3DXRD measurements on a 300 µm α-Fe polycrystalline wire without conical slits. The schematic in Fig. 3 ▸(*a*) depicts the configuration used to acquire diffraction images. The aperture size of the incident four-quadrant slit was set to 20 µm × 20 µm. After aligning the rotation centre (ω), the wire specimen was mounted on the *x*_s_–*y*_s_ stages. At each translation step, the specimen was rotated from 0° to 180° while diffraction images were recorded continuously. The detector acquisition was synchronized with trigger signals generated by a motor controller PM16C-HW2 (Tsuji Electronics) at 3° rotation angle intervals. To increase measurement throughput, we implemented 2 × 2 pixel binning, which enhanced the frame rate and enabled continuous rotation at 20° s^−1^. A dataset for a single *z*_s_ layer was collected within approximately 20 min. The data were acquired at 45 positions along the *X* direction with an interval of Δ*X* = 10 µm, and 60 diffraction images were recorded at each position.

After data acquisition, peak positions were first extracted from the original diffraction images. For each target voxel in the *x*_s_–*y*_s_ layer, diffraction images were selected based on whether the calculated incident beam trajectory intersected the voxel at a given rotation angle and *X*-stage position. The corresponding peak-position data were then assigned to the voxel before multigrain indexing. This method enables reconstruction of the orientation field on a finer voxel grid than the nominal incident beam size by allowing fractional specimen coordinates (Kim *et al.*, 2023*b* ▸). For each voxel, multigrain indexing was performed on the assigned peak-position data to generate multiple orientation matrix candidates. The most probable orientation was then selected as the candidate with the highest completeness factor, *N*′, defined as the fraction of experimentally detected peaks relative to the theoretically expected peaks. After all voxels were processed, the voxelized orientation map was obtained from the orientation matrices assigned to individual voxels. This map was then converted into inverse pole figure (IPF) maps along the *x*_s_, *y*_s_ and *z*_s_ sample directions, denoted as IPF-*x*_s_, IPF-*y*_s_ and IPF-*z*_s_, respectively.

The reconstructed maps of the α-Fe wire are illustrated in Figs. 3 ▸(*b*)–3 ▸(*e*). The nearly random orientations of the constituent grains are evident in the IPF-*x*_s_ [Fig. 3 ▸(*b*)] and IPF-*y*_s_ [Fig. 3 ▸(*c*)] maps, whereas the IPF-*z*_s_ map [Fig. 3 ▸(*d*)] reveals a distinct texture that likely originates from the wire-drawing or fabrication process. As shown in Fig. 3 ▸(*e*), the *N*′ map clearly highlights the grain boundary network in the polycrystalline α-Fe wire.

It is worth noting that the influence of low-intensity beam tails on the reconstructed grain morphology is expected to be limited in this demonstration because the grains are larger than the nominal X-ray beam size. Nevertheless, beam tails may still affect orientation determination near grain boundaries and their influence may become more substantial when the grain size is comparable with or smaller than the nominal beam size (see the supporting information).

### 2.4. EH2 and apparatuses

Fig. 1 ▸(*b*) presents the layout of apparatuses inside EH2, devoted to high-pressure science. The 1500 ton press is permanently placed on the upstream side. Depending on experimental requirements, either the mobile 200 ton press for AE monitoring or the diffractometer with a Paris–Edinburgh press is brought into the downstream side. In addition to the standard four-quadrant slit, a rotational slit system is installed in the most upstream section to spatiotemporally shape the incident X-ray beam. Pseudo-concurrent data acquisition is achieved by sequentially fulfilling the requirements of multiple measurements using spatiotemporally structured X-rays and gating techniques. Combining high-time-resolution diffractometry and radiography enables one to multimodally trace time-dependent structural changes in materials such as a mantle-constituent mineral under the imposed high-pressure and high-temperature conditions by multi-anvil apparatuses. The diffractometer with a Paris–Edinburgh press, which can cover an extensive range of momentum transfers under high-pressure and high-temperature conditions, advances pair distribution function analysis of liquids and amorphous materials.

#### 2.4.1. Large-volume press (MADONNA)

**Figure 4:**
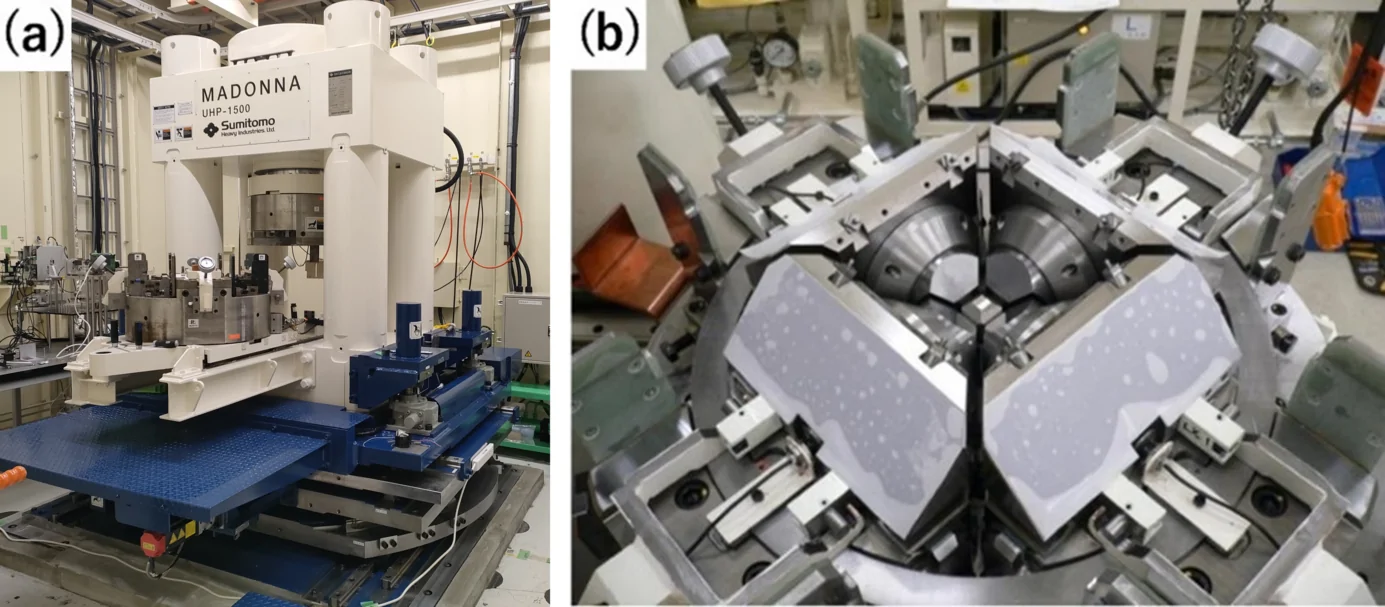
The D-DIA apparatus integrated into the 1500 ton uniaxial press, MADONNA. (*a*) Overall view of the apparatus. (*b*) First-stage anvils and sliding blocks positioned on the MADONNA guide block. Four displacement sensors are mounted on a steel holding ring, which remains unaffected by the deformation of the guide block, to measure anvil displacement.

MADONNA (Multi-Anvil Device On Newer Applications) is designed to investigate (i) phase transitions and accompanying density changes in minerals and rocks at pressures equivalent to those in the Earth’s deep lower mantle (∼50–130 GPa), and (ii) deformation of such materials at pressures of the mantle transition region and the uppermost lower mantle (∼10–30 GPa). The design and dimensions of the uniaxial press are identical to those of the SPEED-MkII (Katsura *et al.*, 2004 ▸) [Fig. 4 ▸(*a*)], while a D-DIA-type guide-block system has been adopted to meet these requirements (Irifune, 2010 ▸) [Fig. 4 ▸(*b*)]. Although the uniaxial press can apply a load of up to 15 MN, the operation of this guide-block system is limited to approximately 13 MN due to strength restrictions of the deformation ram (D-ram).

**Figure 5:**
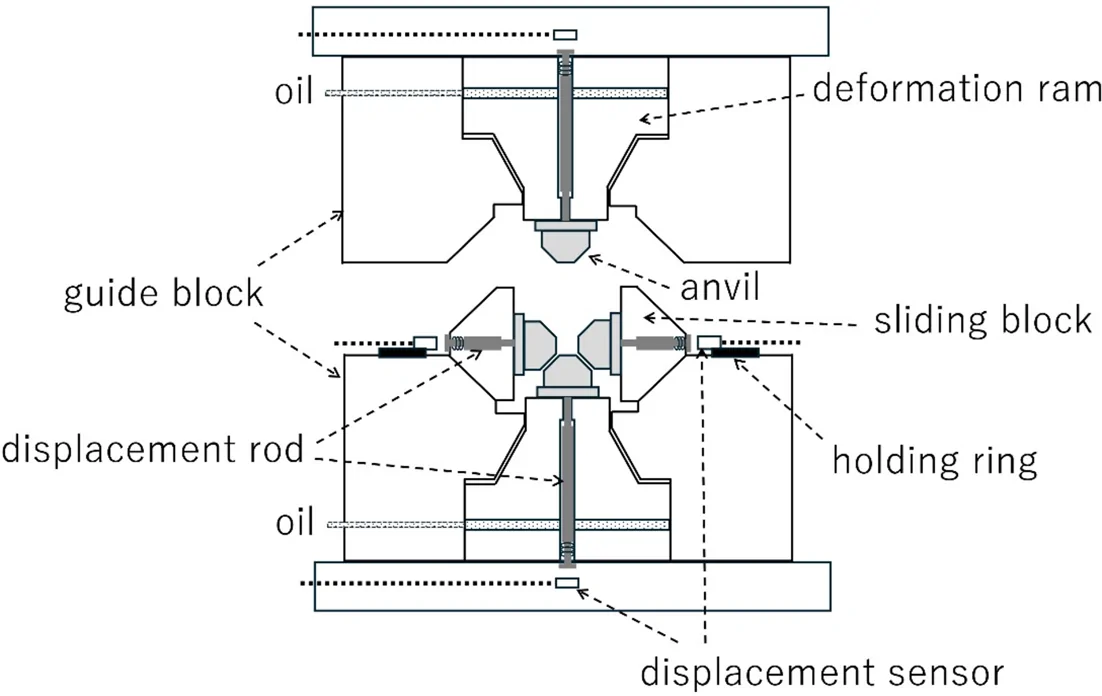
Schematic illustration showing the cross section of the guide blocks, D-rams, sliding blocks and tungsten carbide anvils of the MADONNA apparatus. Anvil positions are monitored by displacement sensors via rods attached to the backsides of the anvils.

For purpose (i), sintered diamond (SD) anvils are employed as the second-stage anvils of the Kawai-type apparatus, where 3D uniform compression of the SD anvil assembly is essential (Katsura *et al.*, 2004 ▸). To determine the positions of the first-stage tungsten carbide anvils mounted on the horizontally moving sliding blocks, displacement rods are attached to the bottoms of the anvils, and their movements are monitored by sensors placed on a holding ring (Fig. 5 ▸). Similarly, the positions of the anvils on the upper and lower D-rams are monitored by displacement rods attached to the backsides of the anvils (Fig. 5 ▸). In this way, the positions of all six first-stage anvils can be simultaneously monitored and adjusted by controlling the D-rams, thereby maintaining a precise cubic space among the six anvils during compression.

**Figure 6:**
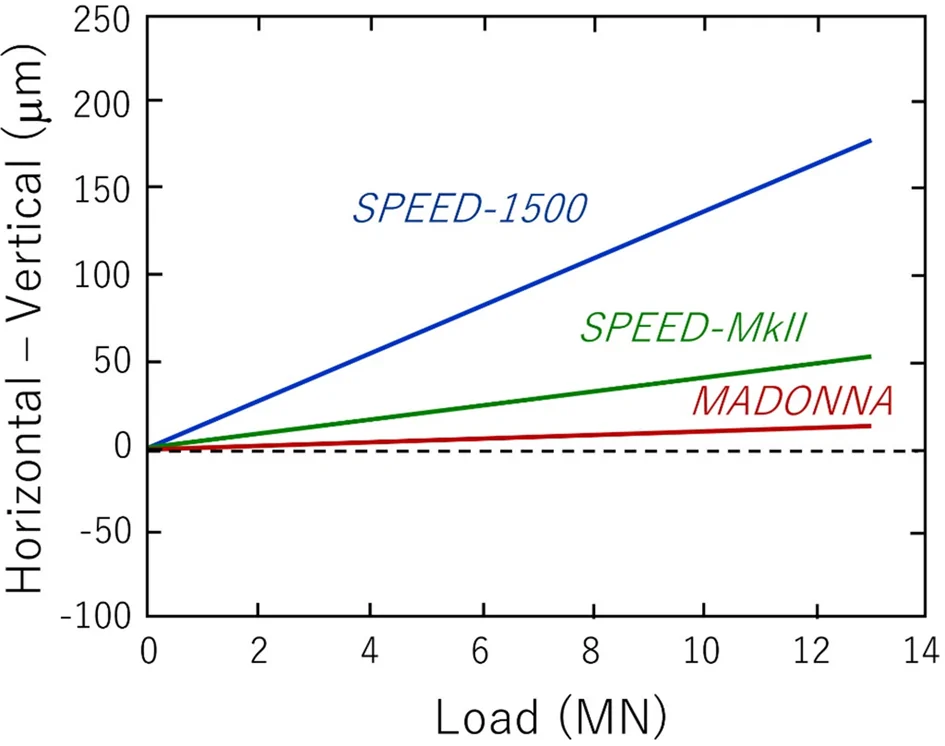
Deformation of the cubic space due to elastic deformation of guide blocks in DIA-type apparatuses at SPring-8. The difference between the horizontal and vertical edge lengths of a compressed copper cube is plotted against the press load. In MADONNA, the difference remains within 10 µm even at a load of ∼10 MN, whereas values reach approximately 150 µm and 50 µm for SPEED-1500 and SPEED-Mk II, respectively (after Katsura *et al.*, 2004 ▸; Irifune, 2010 ▸). Note that these plots represent the verified characteristic operational calibration curves derived directly from the empirical experimental data points compiled in the referenced primary studies.

Although many efforts were made to uniformly compress the second-stage anvil assembly, it later turned out that the design of the MADONNA guide blocks inherently maintains the cubic space under compression without requiring adjustment by the D-rams (Irifune, 2010 ▸). It is well known that the cubic space tends to flatten upon compression due to deformation of the guide blocks, resulting in a substantial increase in the difference between the horizontal and vertical edge lengths of a dummy copper cube used for compression tests (Katsura *et al.*, 2004 ▸) (Fig. 6 ▸). In contrast, using the MADONNA guide blocks, this difference can be suppressed to less than 10 µm even under compression up to ∼10 MN (Irifune, 2010 ▸), which is sufficiently small for high-pressure experiments using SD anvils. Indeed, pressures up to 120 GPa have been successfully generated with SD anvils (Yamazaki *et al.*, 2019 ▸) in combination with the MADONNA guide blocks instead of the conventional DIA-type guide blocks used in the SPEED-Mk II apparatus.

On the other hand, the displacement rods installed in the upper and lower D-rams are fully utilized for purpose (ii) mentioned above. These rods are pressed against the bottoms of the anvils by spring coils, thereby preventing deformation of the D-ram pistons from affecting the displacement of the sample cell. Numerous high-pressure deformation experiments (*e.g.* Ohuchi *et al.*, 2011 ▸; Ohuchi *et al.*, 2014 ▸) have been successfully conducted off-line using the MADONNA guide blocks. The D-111-type guide block, a deformation apparatus optimized for the Kawai-type (6–8 type) multi-anvil press, was recently developed (Hunt *et al.*, 2014 ▸). This guide block can also be implemented in the MADONNA press, enabling experiments with pressures up to 30 GPa (Tsujino *et al.*, 2022 ▸). Nevertheless, X-ray imaging with intense synchrotron radiation enables direct measurement of sample deformation without relying on the displacement of the anvils (*e.g.* Kawazoe *et al.*, 2011 ▸; Ohuchi *et al.*, 2015 ▸; Nishihara *et al.*, 2018 ▸).

The MADONNA has been installed on a newly designed press stage at EH2 for *in situ*X-ray observations under high-pressure and high-temperature conditions. The stage system of MADONNA adheres to the fundamental design described by Katsura *et al.* (2004 ▸) for the SPEED-MkII and consists of five axes: *Y*1, κ, *X*, *Y*2 and *Z*. Although the required ranges of motion and positioning accuracy are comparable with those of the SPEED-MkII stage system, translation motion must be much faster in order to reduce measurement overheads that lead to a significant loss of photons, as in 3D mapping. Specifically, the maximum speeds in the horizontal (*X* and *Y*2) directions are almost an order of magnitude higher than those of the SPEED-MkII, whereas the vertical (*Z*) and rotational (κ) stages exhibit a roughly threefold increase in speed compared with the SPEED-MkII.

The incident sliding blocks and first-stage WC anvils have 10° and 7° slits, respectively. The receiving sliding blocks and first-stage WC anvils are equipped with a 10° cone, ensuring a clear image field around the sample and enabling 2D-XRD (where XRD is X-ray diffraction) acquisition. For the second-stage anvil on the downstream side, either a WC anvil with a cone-shaped cutout, or a c-BN or SiC binder SD anvil that is relatively transparent to X-rays, is specifically used to acquire 2D-XRD images. To observe a clear sample XRD pattern without the use of a receiving slit, the gasket and Cr_2_O_3_-doped MgO pressure medium in the X-ray path are typically replaced with an amorphous boron + ep­oxy resin mixture.

**Figure 7:**
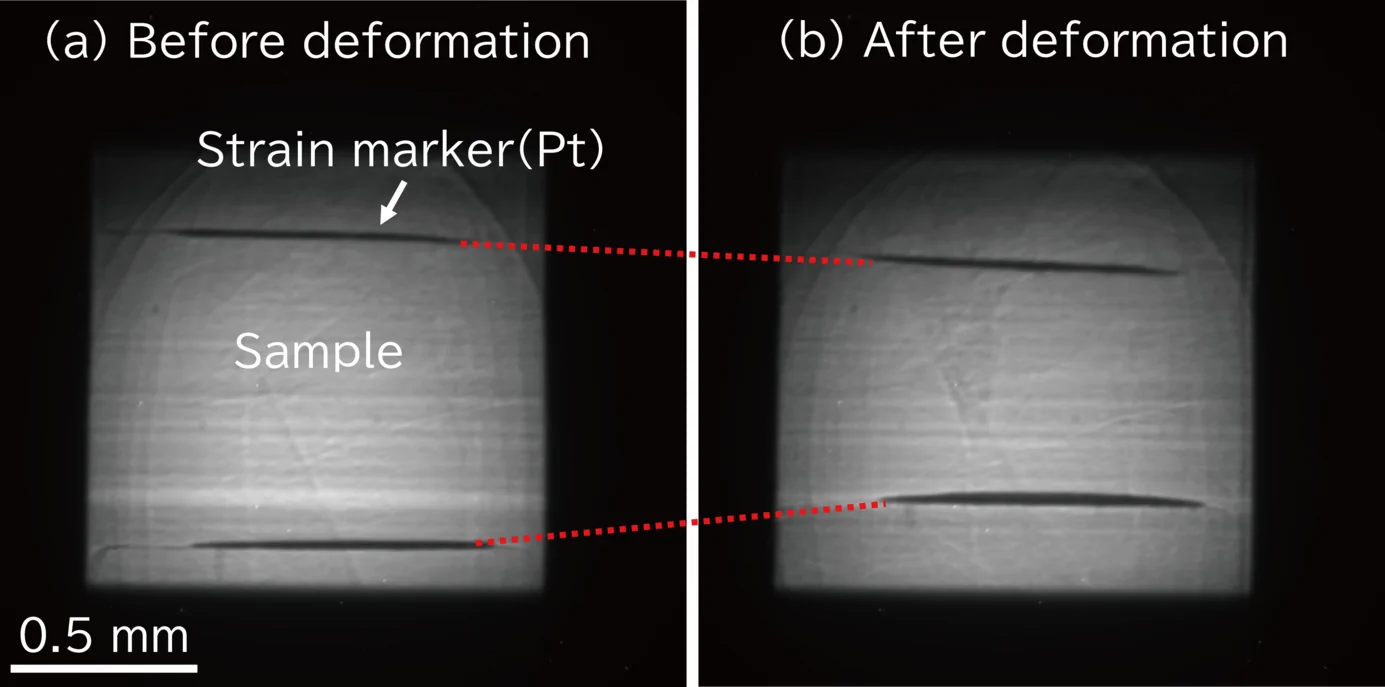
X-ray radiographs of uniaxial deformation experiments of ortho-enstatite using the MADONNA press. (*a*) Before deformation. (*b*) After deformation. Ortho-enstataite aggregate sample enclosed in Fe foil was located between Pt strain markers. After uniaxial deformation, sample length was shorter and total strain reached 28%.

**Figure 8:**
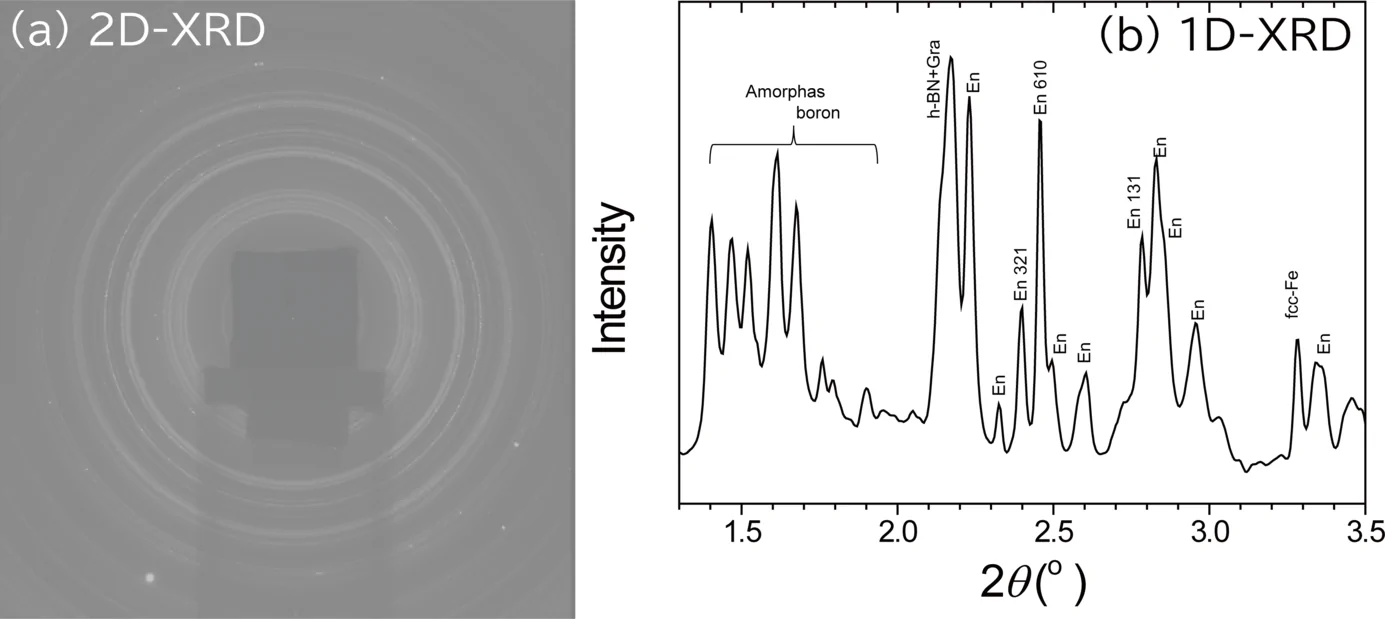
XRD pattern of enstatite sample during deformation experiments at 1573 K and 1.5 GPa. (*a*) 2D-XRD image. (*b*) Converted 1D-XRD pattern. The diffraction peaks of amorphous boron were observed down to approximately 1.8°. During deformation, diffraction peaks of the sample ortho-enstatite, the surrounding h-BN and Fe foils, and the graphite heater were observed. In this study, stress and pressure were analysed using diffraction lines 321, 610 and 131, which are particularly easy to separate as enstatite diffraction lines.

A 150 µm-thick LUAG:Ce scintillator in combination with a CMOS camera is used for imaging measurements. To minimize the blind region on a 2D-XRD image, the camera body is positioned outside the 10° cone using an 8× telecentric lens with a long focal length. The CMOS camera (C14440-20UP, Hamamatsu Photonics K.K.) has 2304 × 2304 pixels with 6.5 µm per pixel, resulting in a theoretical field of view of 1.87 mm × 1.87 mm, or 0.813 µm per pixel. Fig. 7 ▸ shows X-ray radiography before and after deformation experiments using a 6–6-type deformation cell. Strain markers of Pt were clearly visible without correction, allowing for determination of sample length and strain. For 2D-XRD measurements, a flat-panel detector (Radicon-2022, Teledyne Rad-icon Imaging) is employed, with each element measuring 99 µm and having a size of 220 mm × 200 mm. This detector was installed approximately 1.56 m from the sample in this study. This allowed for observation of a full circle up to 2θ = 3.7° (corresponding to 1.92 Å) and diffraction up to 5.4° in 2θ (corresponding to 1.315 Å). Fig. 8 ▸ shows a 2D-XRD image and its corresponding 1D-XRD profile. Outside the central shadow region on the 2D-XRD image, high-intensity diffraction signals from the sample were clearly observable across the entire azimuthal angle range. The centre of Debye rings was determined from a weak direct beam spot that was attenuated not only by the scintillator and half-mirror but also by Pb and Ta foils.

In MgSiO_3_ compositions, protho-enstatite is a stable phase under high-temperature conditions below 1 GPa. The viscosity of ortho-enstatite, which is stable at pressures above 1 GPa up to 9 GPa, remains poorly constrained despite being one of the major minerals in the Earth’s upper mantle. Experimental investigations of ortho-enstatite under wet conditions have revealed that, despite having a relatively large particle size (∼6 µm), diffusion creep is dominant and its temperature dependence (∼200 kJ mol^−1^) is quite small (Zhang *et al.* 2017 ▸). This contrasts significantly with olivine, the most abundant mineral in the upper mantle, suggesting that the viscosity constraint of ortho-enstatite is important for accurately modelling the rheology of the upper mantle.

**Figure 9:**
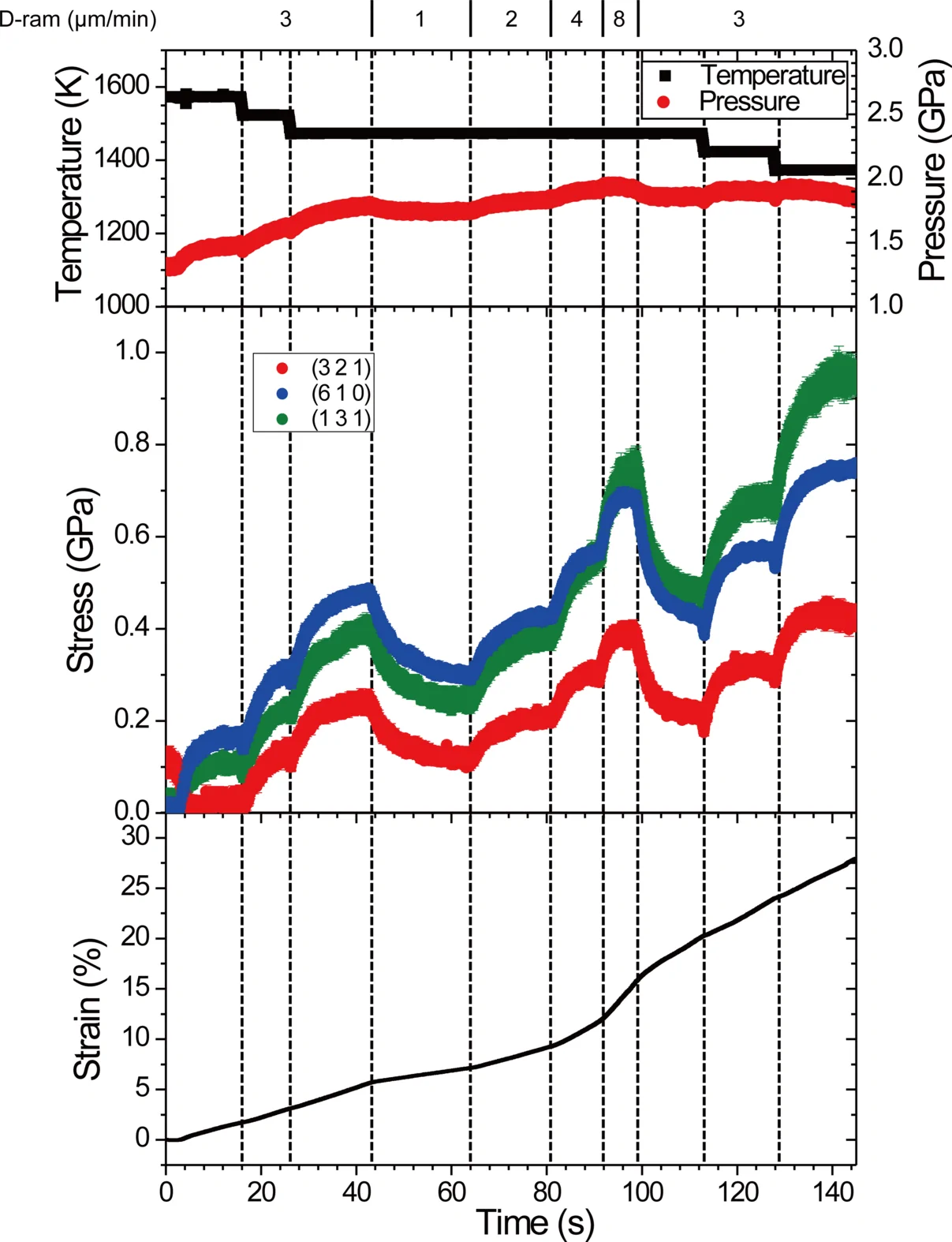
Temperature, pressure, stress and strain data plotted against time at 0.45 MN under nominally dry conditions. Pressures were determined by the equation of state of ortho-enstatite. Stress values were obtained from three diffraction peaks of enstatite (red: 321; blue: 610; green: 131). Temperature, pressure and strain are shown by black squares, red circles and black circles, respectively. After initial deformation at 1573 K and confirmation of steady-state deformation, the steady-state deformation was observed while cooling in 50 K steps. Subsequently, the strain rate dependence was measured by changing the D-ram stroke speed from 1 µm min^−1^ to 8 µm min^−1^, and then the temperature dependence was measured by further changing the temperature. Finally, total strain reached 28%.

To demonstrate the performance of the MADONNA press, high-temperature and high-pressure deformation experiments were conducted on MgSiO_3_ ortho-enstatite under nominally dry conditions by Fe foil using a 100 keV pink beam. A 6–6-type cell assembly with a truncation edge length (TEL) of 6 mm was employed. The WC anvil with a cone-shaped cutout was used for the second-stage anvil on the downstream side. The sample was initially pressurized to a main-ram load of 0.45 MN, after which the temperature was increased to 1573 K. Once the target temperature was attained, the sample underwent deformation at constant stroke rates of the D-rams. Sample strain was determined by monitoring changes in specimen length through X-ray absorption imaging, as illustrated in Fig. 7 ▸. Meanwhile, differential stress was estimated from the lattice strain reflected as the distortion of the Debye rings in the 2D-XRD pattern collected using an X-ray flat-panel detector. Fig. 8 ▸(*a*) displays a raw 2D-XRD image, in which a diffraction pattern is observable at 2θ angles above 1°. Stress and pressure were calculated using the 321, 610 and 131 enstatite diffraction peaks, which were distinctly separable from other diffraction peaks on the corresponding 1D-XRD profile. For the stress estimation, the elastic constants of single-crystal orthopyroxene reported by Kumazawa (1969 ▸) were utilized. The sample pressure was calculated based on the equation of state for MgSiO_3_ ortho-enstatite (Zhao *et al.*, 1995 ▸). Fig. 9 ▸ illustrates the time evolution of strain, differential stress and pressure. The results clearly show that variations in strain and stress are well correlated with the D-ram speeds and temperature. These data are currently being analysed to further elucidate the deformation mechanisms of MgSiO_3_ enstatite. Although this demonstration was performed under relatively modest loading conditions, further experiments in higher-pressure regimes are envisioned for future research endeavours.

#### 2.4.2. Mobile multi-anvil apparatus ‘Hyaku-shiki’

**Figure 10:**
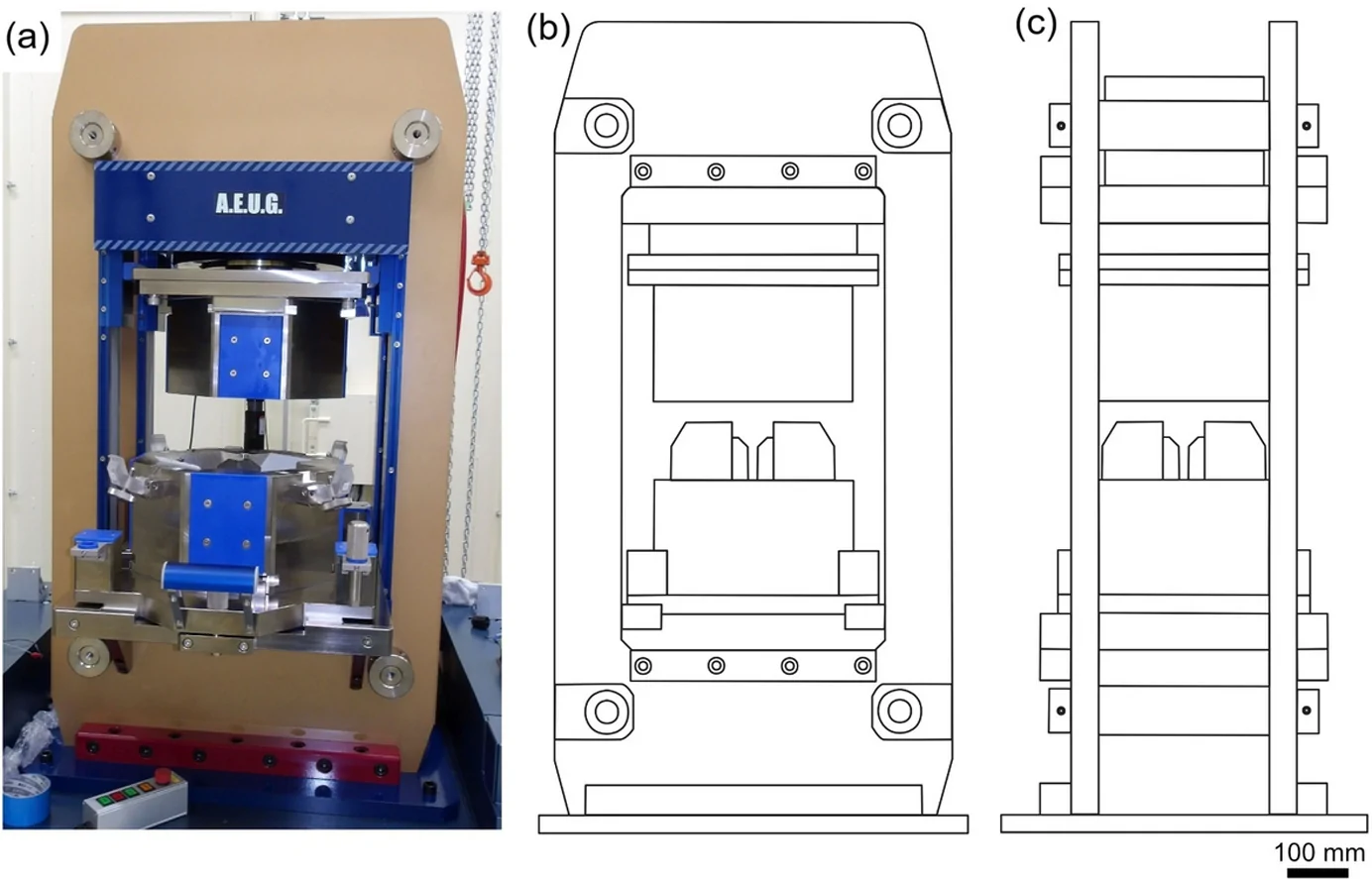
(*a*) The 200 ton mobile press Hyaku-shiki with the D-DIA guide blocks. (*b*, *c*) Schematic illustrations of the Hyaku-shiki press. Views perpendicular and parallel to the X-ray path are shown in (*b*) and (*c*), respectively.

A mobile DIA-type multi-anvil apparatus (Hyaku-shiki) was developed for *in situ* deformation experiments on minerals and rocks at high pressures and high temperatures using synchrotron X-rays (Fig. 10 ▸). The total weight of the Hyaku-shiki press and its positioning system is 5 ton, allowing for manual transportation using a hand pallet truck. The newly designed load-bearing frame for the Hyaku-shiki consists of two thick steel plates (Fig. 10 ▸) and maintains a maximum press load of 200 tonf while providing an open space with a width of 280 mm on the upstream and downstream sides of the guide blocks. In D-DIA-type guide blocks, the first-stage WC anvils at the downstream side are equipped with a 5° cone-shaped cutout, ensuring a clear image field around the sample and enabling 2D-XRD acquisition. This wide open space can accommodate not only a DIA-type guide block but also small apparatus, such as a rotational Drickamer-anvil apparatus for X-ray computed microtomography (Wang *et al.*, 2005 ▸). The lightweight design of the Hyaku-shiki press allows the five-axis positioning system (*Y*1, κ, *Y*2, *X* and *Z*) to employ stepping motors, thereby enabling AE measurements under low-noise environments. The maximum speeds are 2 mm s^−1^ for the horizontal axes, 0.14 mm s^−1^ for the vertical axis and 0.3° s^−1^ for the rotational axis, ensuring rapid position alignment.

**Figure 11:**
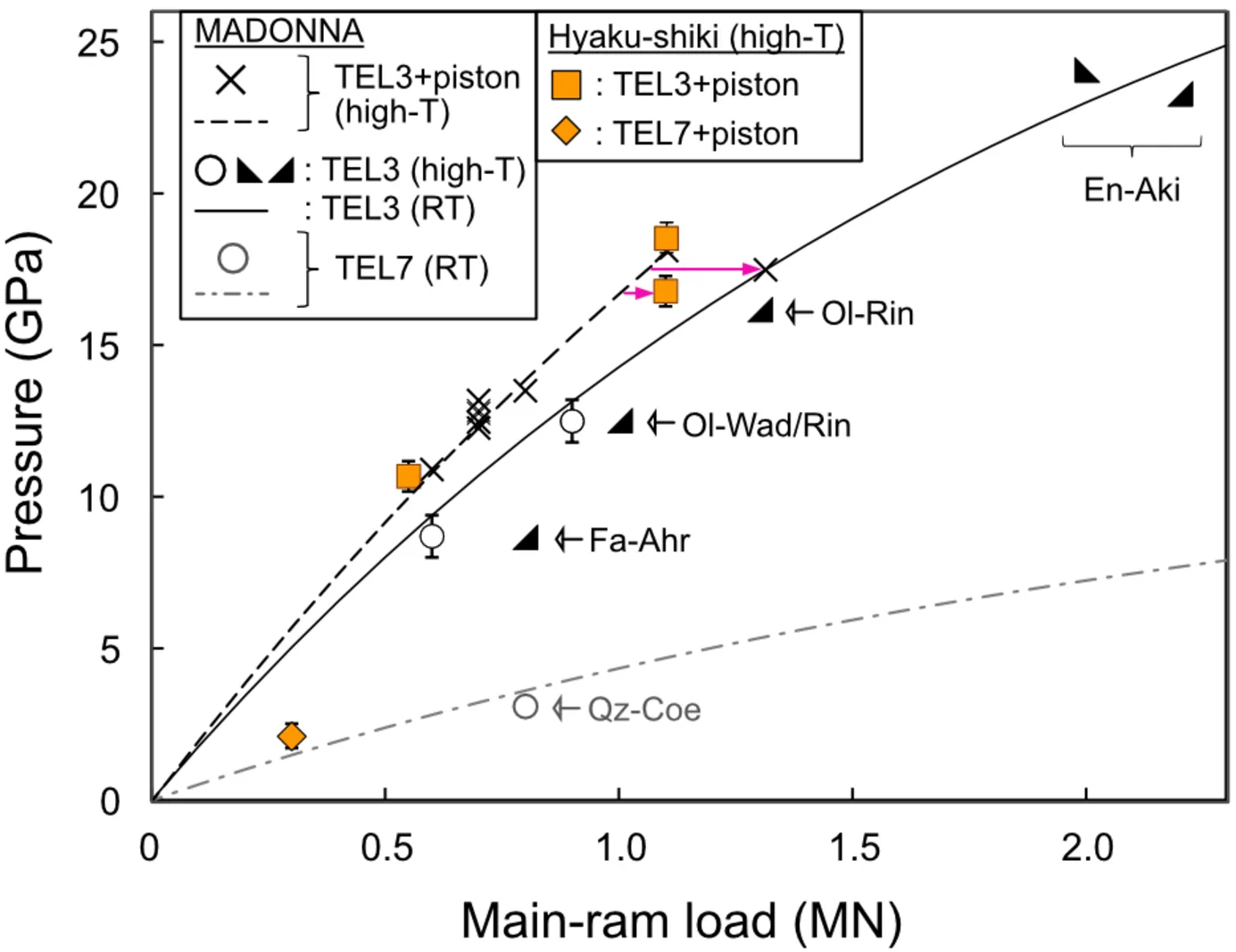
Relationships between the applied main-ram load and the generated pressure for MA 6–6 cell assemblies in the Hyaku-shiki press. Solid squares and diamonds represent pressures generated using second-stage anvils with TEL 3 (at 1120–1470 K) and TEL 7 (at 1150 K), respectively. Pressures were determined by the equation of state of olivine. Compression curves were determined from a series of pressure-calibration runs in the MADONNA press. Dot-dashed curve: TEL 7 and the cubic pressure medium with an edge length (CEL) of 11 mm at room temperature. Solid curve: TEL 3/CEL 5 at room temperature. Dashed curve: TEL 3/CEL 5 at 1120–1570 K. The black symbols represent the pressures generated in the MADONNA press. Crosses: pressures determined by the equation of state of olivine (note that the efficiency of pressure generation was improved because the olivine sample was sandwiched by two hard-alumina pistons). Circles and triangles: determined by the phase assemblages in the recovered samples. The starting material was powders of quartz, Fe_2_SiO_4_ fayalite, (Mg_1.8_,Fe_0.2_)SiO_4_ olivine or (Mg_0.9_,Fe_0.1_)SiO_3_ ortho-enstatite. Qz: quartz; Coe: coesite; Fa: fayalite; Ahr: ahrensite; Ol: olivine; Wad: wadsleyite; Rin: ringwoodite; En: ortho-enstatite; Aki: akimotoite.

The D-DIA apparatus, consisting of a main-ram and two built-in hydraulic rams (D-rams) in the upper and lower guide blocks (Wang *et al.*, 2003 ▸), enables *in situ* deformation runs under high pressures. The Hyaku-shiki D-DIA guide blocks, approximately 350 mm in diameter, have six first-stage anvils with a TEL of either 27 or 50 mm, which are compatible with various types of MA6-6 (Nishiyama *et al.*, 2008 ▸; Kawazoe *et al.*, 2010 ▸) and Kawai-type (Kawai *et al.*, 1973 ▸) cell assemblies. Fig. 11 ▸ shows the results of the pressure calibration experiments as a function of main-ram load. The efficiency of pressure generation was comparable with that in the MADONNA press (Ohuchi *et al.*, 2022 ▸), and it improved further when the sample was sandwiched by two hard-alumina pistons during deformation runs (dashed curve in Fig. 11 ▸). The sample pressure reached ∼19 GPa at 1.1 MN with TEL 3 mm and deformation pistons at 1120 K. A few results at 1.1–1.3 MN deviated from the compression curve for the cell assembly with TEL 3 anvils and deformation pistons (shown by pink arrows in Fig. 11 ▸) due to pre-annealing of the sample at 1470 K and 0.55 MN. No such deviation was observed in the case of pre-annealing at 1370 K and 0.55 MN (the square plotted at ∼19 GPa and 1.1 MN in Fig. 11 ▸).

To illustrate the capabilities of the Hyaku-shiki press, a preliminary high-pressure deformation experiment was conducted on (Mg,Fe)_2_SiO_4_ olivine, which is the most abundant mineral in the Earth’s mantle and in subducted slabs where numerous deep earthquakes have been observed. Brittle fracture deformation of olivine is believed to contribute to the occurrence of deep earthquakes, making *in situ* observation and simultaneous AE measurements during deformation experiments essential for understanding their mechanism. A semi-sintered cube of cobalt-doped magnesia with an edge length of 5 or 11 mm was used as the pressure medium and was surrounded by six second-stage anvils with a TEL of 3 or 7 mm. Five second-stage anvils were made from tungsten carbide. An X-ray-transparent second-stage anvil (made from SD or cubic boron nitride) was placed on the downstream side. The cell assembly was first pressurized hydro­statically up to a main-ram load of 0.3 MN, and then the temperature was raised to 1180 K. Once the target temperature was attained, the sample underwent triaxial compression at a constant stroke rate (10 µm min^−1^) of the upper and lower first-stage anvils while AEs were monitored. 2D radial diffraction patterns were acquired using a cadmium telluride imaging detector (WidePix 5 × 5) with an exposure time of 0.4 s. Readers are referred to Ohuchi *et al.* (2025 ▸) for further details on experimental procedures.

**Figure 12:**
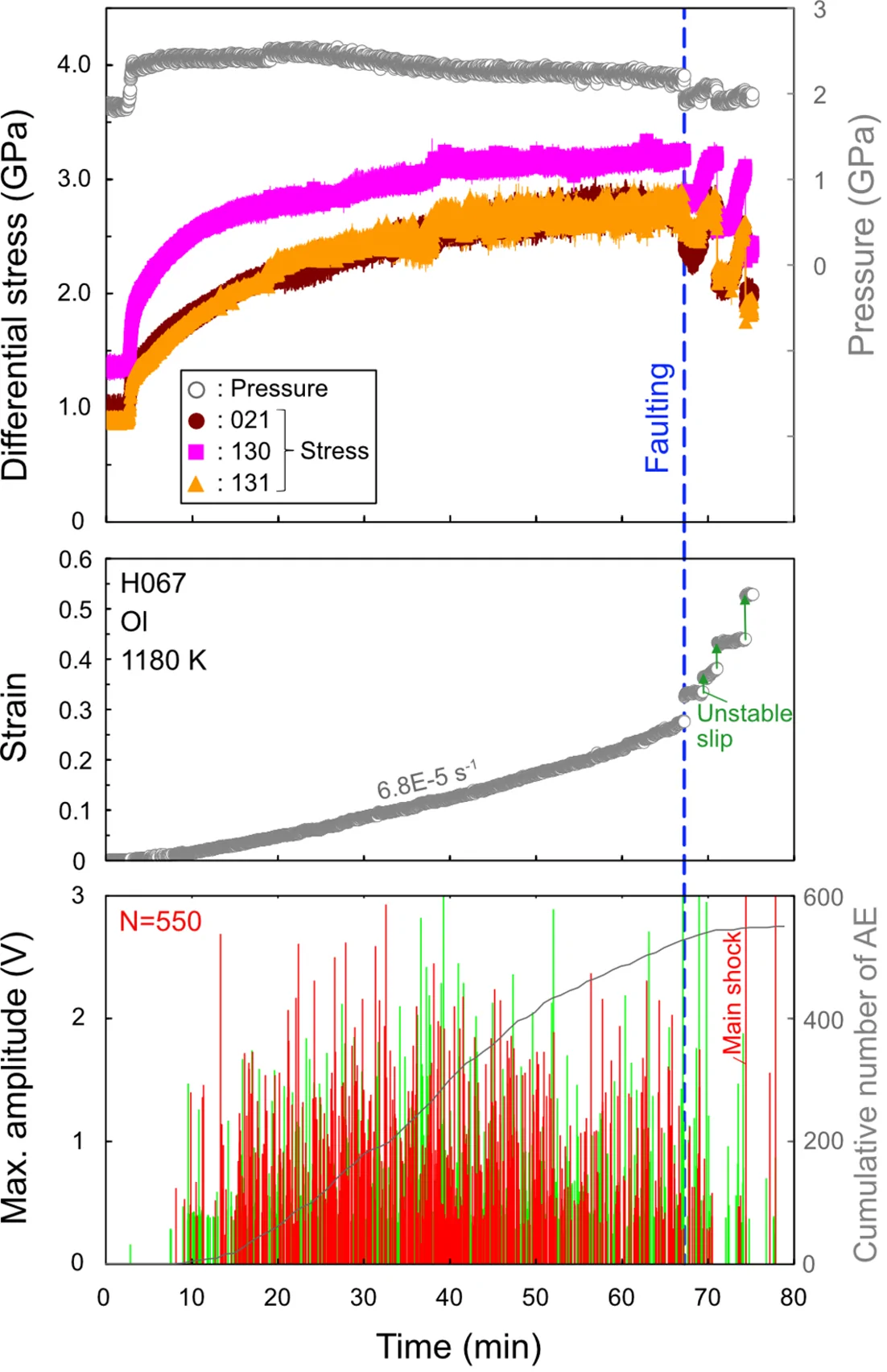
Mechanical (pressure, stress, strain) and acoustic records (maximum amplitude of each event and cumulative number of events) in olivine deformed at 1180 K under nominally dry conditions. Pressures were determined by the equation of state of olivine (Liu *et al.*, 2005 ▸). Stress values were obtained from three diffraction peaks of olivine (circles: 021; squares: 130; triangles: 131). Pressure and strain are shown by grey open circles. The cumulative number of AEs (grey line) and amplitudes (red lines: AEs from inside the sample; green lines: outside the sample) are plotted against time. The total number of AE events from the inside of the sample (*N*) is also shown. AE events were collected with six piezoceramic lead-zirconate titanate transducers (narrow-band type with the resonant frequency of ∼4 MHz) that were attached to the rear side of each second-stage anvil.

Fig. 12 ▸ shows the time evolution of mechanical and acoustic records in the olivine sample deformed at pressures ranging between 1.9 and 2.5 GPa and at a temperature of 1180 K. The uncertainties in pressure and stress values were within ±0.1–0.2 GPa. The use of intense X-rays allowed us to evaluate short-duration events that proceed within seconds, such as faulting (at 67 min in Fig. 12 ▸) and unstable slips of the fault associated with stress drops (at 69, 71 and 74 min in Fig. 12 ▸). Over 500 AEs were radiated from inside the sample (red lines in Fig. 12 ▸). Prior to the onset of faulting, many AE events (maximum amplitude < 3 V) and an acceleration in the AE rate were observed. Following the occurrence of faulting, the AE rate decreased, and a few large AE events (*i.e.* main shocks) were radiated.

#### 2.4.3. High-pressure pair distribution function measurement in the Paris–Edinburgh press

Understanding structural changes in liquids and amorphous materials under *in situ* high-pressure and high-temperature conditions is fundamental to scientific disciplines such as physics, chemistry, geoscience and materials science, as well as technological applications. Advances in investigations of pressure-induced structural changes in liquids and glasses have been stimulated through the realization of X-ray pair distribution function (PDF) measurements combined with high-pressure experiments, such as large-volume presses (Mezouar *et al.*, 2002 ▸; Kono *et al.*, 2014 ▸; Yu *et al.*, 2019 ▸; Henry *et al.*, 2022 ▸) and diamond anvil cells (Shen *et al.*, 2003 ▸; Sato *et al.*, 2010 ▸; Prescher *et al.*, 2017 ▸). However, a fundamental challenge in high-pressure PDF measurements lies in the limited available momentum transfer (*Q*) range in the structure factor [*S*(*Q*)], which constrains the resolution of the PDF [*g*(*r*)] derived via Fourier transform. To overcome this limitation with the aid of high-energy X-rays, the high-pressure PDF measurement setup has been deployed in EH2, enabling the measurement of the *S*(*Q*) of liquids and glasses at a *Q* range up to 27.8 Å^−1^. This section provides a brief introduction to the setup and the available ranges of pressure and temperature. Details of the high-pressure PDF measurement are described by Kono *et al.* (2024 ▸).

**Figure 13:**
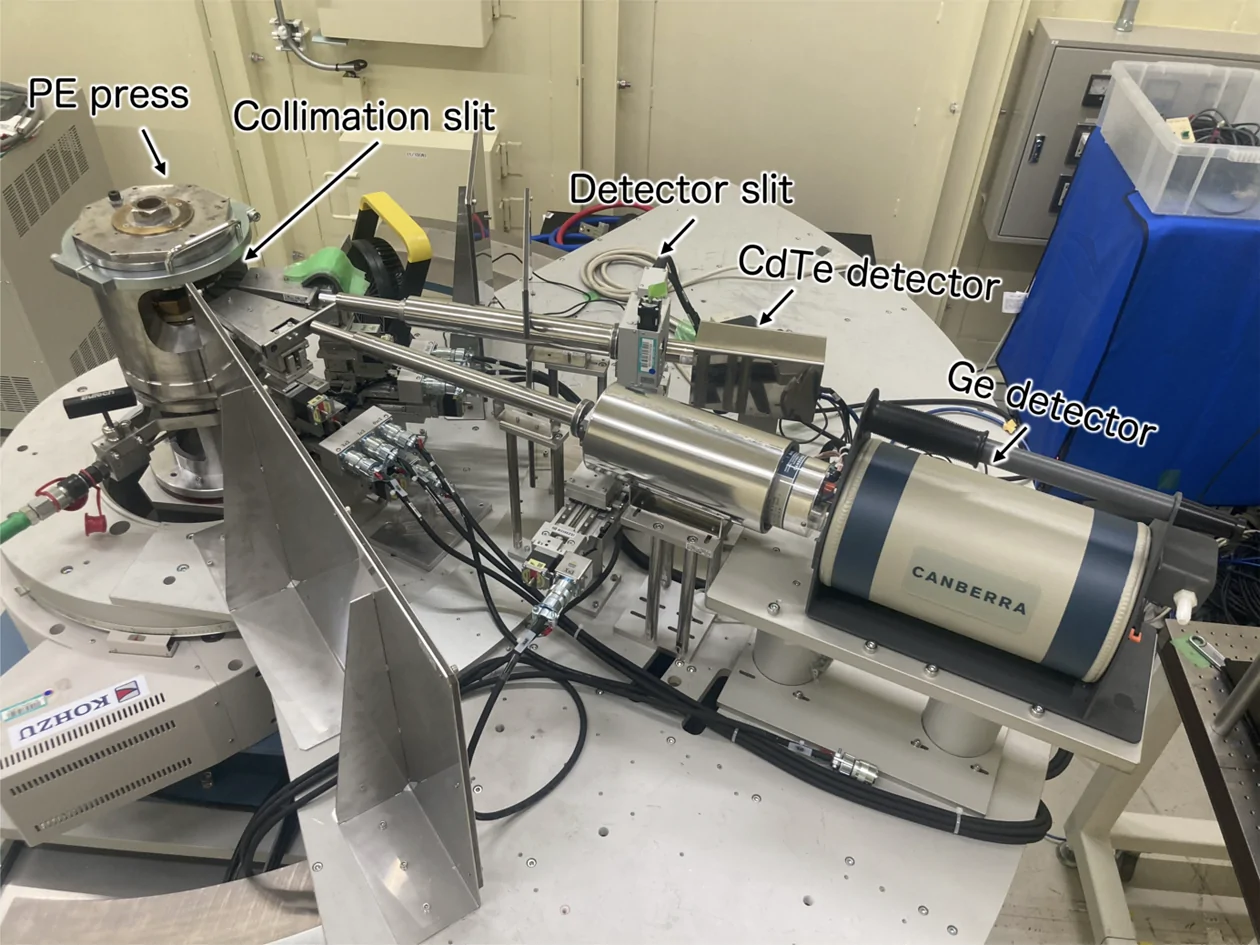
Diffractometer setup for *in situ* high-pressure PDF measurement combined with a Paris–Edinburgh press at EH2.

**Figure 14:**
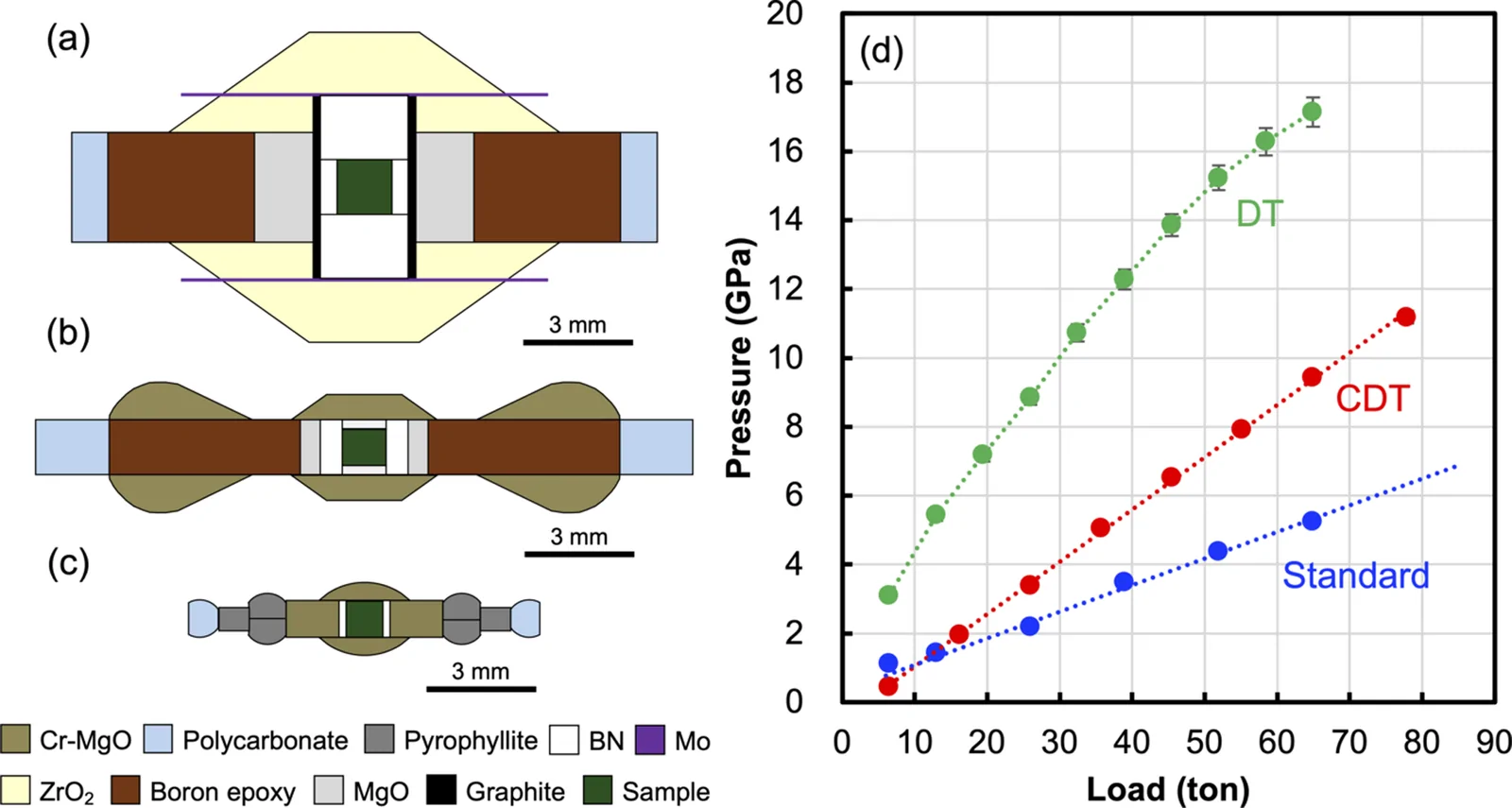
Paris–Edinburgh press cell assemblies and performance. (*a*) Design of the standard cell for high-pressure and high-temperature experiments, (*b*) cupped-Drickamer-toroidal (CDT) cell assembly (Kono *et al.*, 2014 ▸) for room-temperature experiments up to 12 GPa, (*c*) newly developed double-toroidal (DT) cell assembly for higher-pressure experiments. (*d*) Pressure generation curves for the standard (blue), CDT (red) and DT (green) cell assemblies.

Fig. 13 ▸ shows the experimental setup. The size of the X-ray beam is adjusted by the incident slit located ∼1.2 m upstream of the sample position. A transmission-type Si photodiode (crystal thickness 50 µm) is placed just after the incident slit to monitor the intensity of the incoming X-rays. High-energy X-ray diffraction measurements are conducted using a combination of two point detectors: an X-123 CdTe detector (Amptek) for low 2θ angles and a Ge detector (Mirion Technologies) for high 2θ angles. The Ge detector is chosen specifically for the high-2θ range due to its detection efficiency being approximately twice that of the 1 mm-thick CdTe detector at 100 keV, making it indispensable for capturing weak scattering signals at high *Q*. These detectors are placed at a separation angle of 15° to collect XRD data at 2θ angles up to 31.8°. Each detector is equipped with a double-slit collimation setup (collimation slit and detector slit) to collect weak scattering signals from liquids and amorphous materials without background signals from surrounding high-pressure-cell components (Fig. 14 ▸).

High-pressure experiments are conducted using a Paris–Edinburgh press, which has a wide opening in the horizontal plane (Fig. 13 ▸). The wide opening enables high-energy XRD measurements at wide 2θ angles for obtaining *S*(*Q*) data over a wide range of *Q*. Three types of high-pressure cells are used depending on the target pressure and temperature (Fig. 14 ▸). The standard cell assembly [Fig. 14 ▸(*a*)] can withstand experiments up to 7 GPa and 2300 K, enabling PDF measurement on both glasses and melts. The cupped-Drickamer-toroidal (CDT) cell assembly [Fig. 14 ▸(*b*)] is used for room-temperature experiments up to 12 GPa. In addition, we recently developed a double-toroidal (DT) type cell assembly shown in Fig. 14 ▸(*c*). Pressure generation up to 17 GPa is possible with the DT cell assembly based on the dimple diameter (DD) = 2.5 mm cell design of Hattori *et al.* (2019 ▸). In the new DT cell assembly, WC is employed as the anvil material instead of the SD anvil used in the original study.

The combination of the 100 keV pink beam and the large-volume Paris–Edinburgh press experiment at BL15XU, SPring-8 enables *S*(*Q*) measurements in the *Q* range up to 27.8 Å^−1^, which significantly widens the accessible *Q* range compared with previous high-pressure studies conducted at other facilities. High-pressure PDF measurements by angle-dispersive XRD using area detectors at ESRF ID27 and APS 13IDC allow shorter acquisition times of roughly 5–10 min, while their typical *Q* ranges reach up to ∼10–12 Å^−1^ (*e.g.* Morard *et al.*, 2018 ▸; Yu *et al.*, 2019 ▸). Combined angle- and energy-dispersive setups at SOLEIL PSICHE report *S*(*Q*) measurements at a *Q* range up to ∼10–13 Å^−1^ in approximately 20 min (*e.g.* King *et al.*, 2022 ▸; Henry *et al.*, 2022 ▸). In contrast, multi-angle energy-dispersive measurement setups at APS 16BMB can attain slightly higher *Q* ranges of ∼12–15 Å^−1^, but their acquisition times are substantially longer, exceeding 2–3 h due to limited X-ray flux (*e.g.* Hudspeth *et al.*, 2018 ▸; Shibazaki *et al.*, 2020 ▸). The setup of BL15XU achieves *S*(*Q*) measurements at an extensive *Q* range up to 27.8 Å^−1^ in a reasonably short acquisition time of 30 to 60 min. This capability almost doubles the accessible *Q* range for previous high-pressure angle-dispersive measurements and opens new possibilities for investigating the detailed structural features of liquids and amorphous materials under *in situ* high pressures and temperatures.

**Figure 15:**
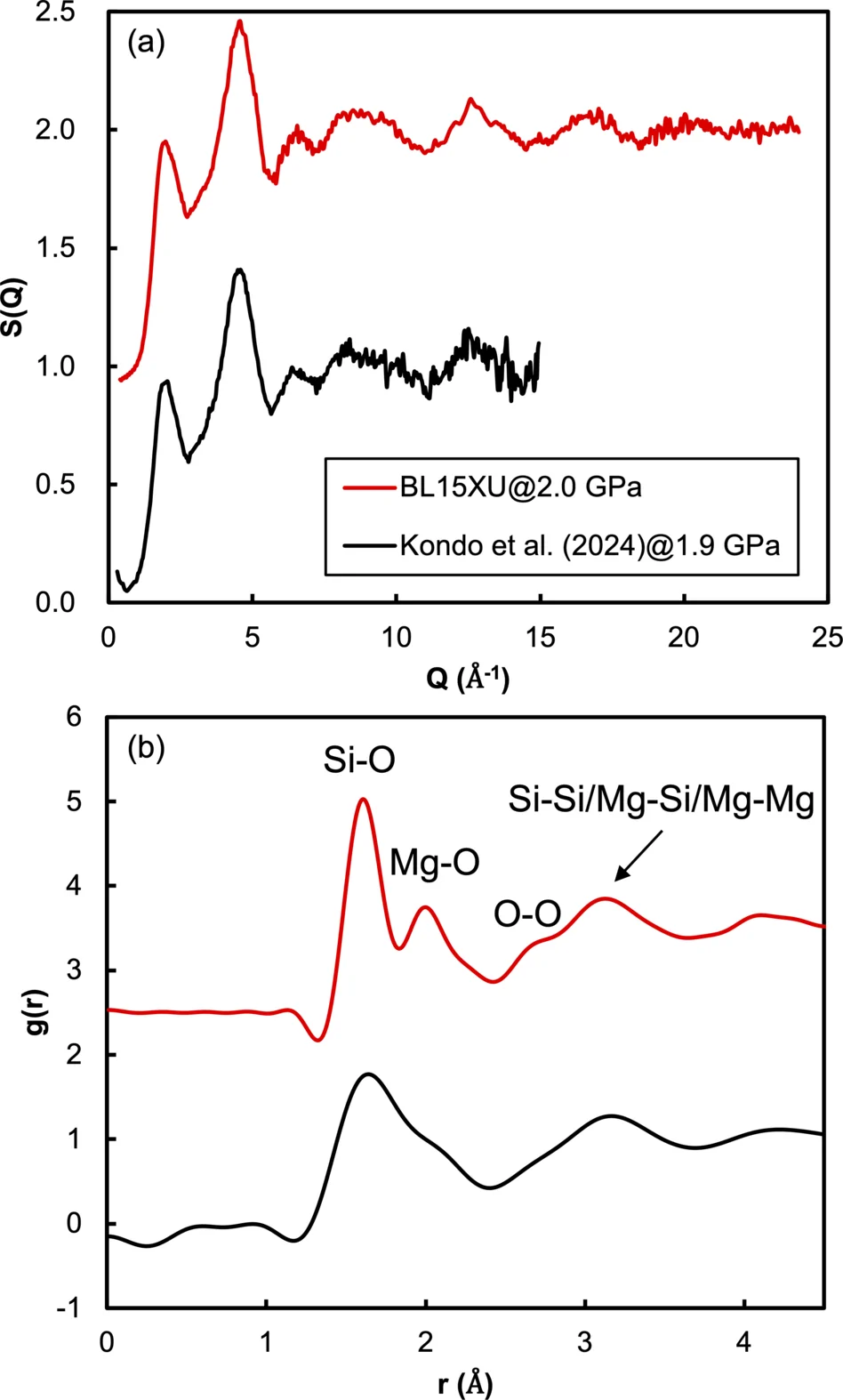
Structural analysis of MgSiO_3_ glass at ∼2 GPa. (*a*) Structure factor *S*(*Q*) and (*b*) PDF *g*(*r*) measured at BL15XU, compared with the result from Kondo *et al.* (2024 ▸). The *S*(*Q*) and *g*(*r*) data from BL15XU are displayed with vertical offsets of 1.0 and 2.5, respectively. This comparison demonstrates the capability of the BL15XU setup to access a significantly wider *Q* range (up to ∼24 Å^−1^). Consequently, the main conclusion drawn from this extended *Q* range is a highly improved real-space resolution in *g*(*r*), which enables the clear separation and distinct observation of the closely spaced Si–O and Mg–O peaks.

Fig. 15 ▸ shows representative examples of *S*(*Q*) and the PDF *g*(*r*) for MgSiO_3_ glass under high-pressure conditions of ∼2 GPa at room temperature. For comparison, the figure also shows the results of a previous study by Kondo *et al.* (2024 ▸), which revealed *S*(*Q*) up to *Q* = 15 Å^−1^ using an X-ray energy of 37.4 keV. A 100 keV pink beam allowed us to determine *S*(*Q*) up to *Q* = 24 Å^−1^ within a relatively narrow 2θ range. The wide *Q* range improved the real-space resolution of *g*(*r*), as the Si–O and Mg–O peaks in MgSiO_3_ glass are clearly resolved [Fig. 15 ▸(*b*)].

#### 2.4.4. Auxiliary device

**Figure 16:**
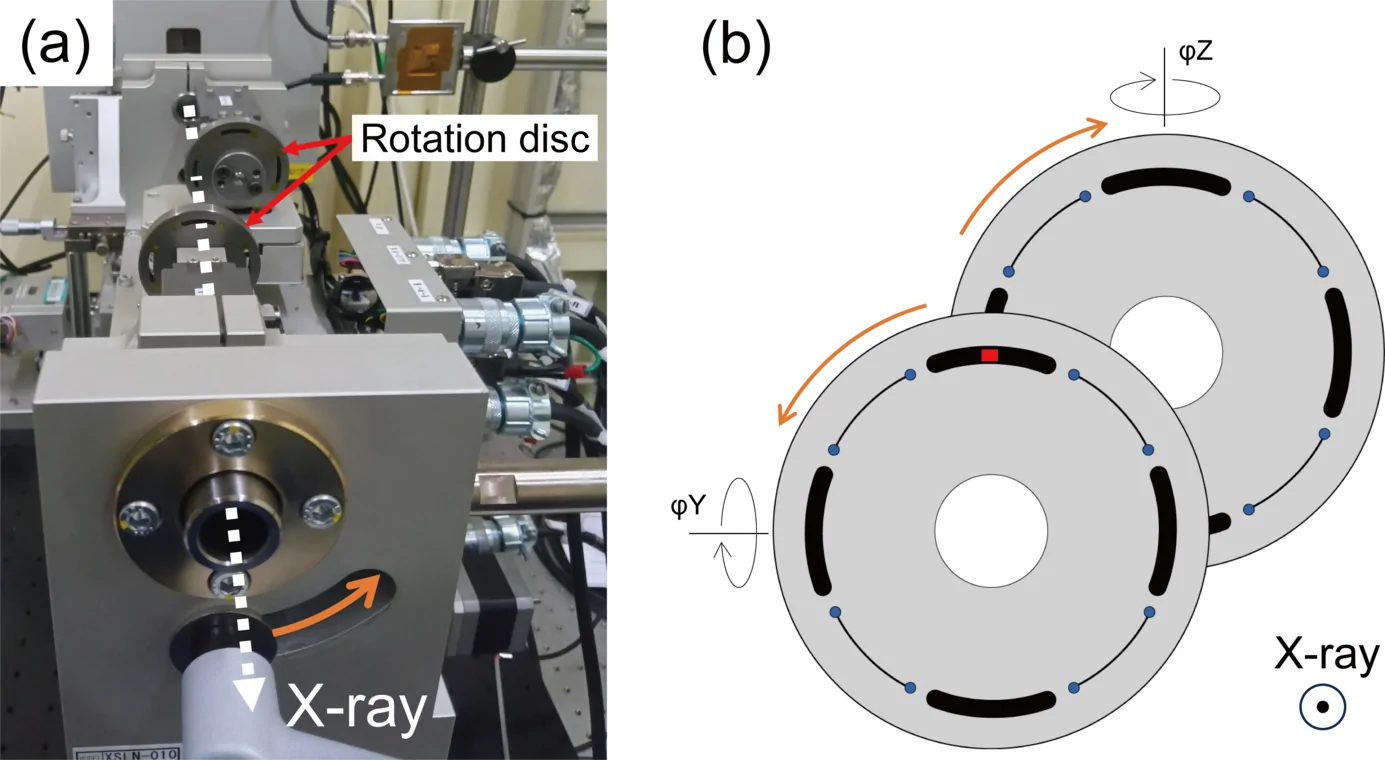
Rotational slit system in EH2. (*a*) A white dashed arrow and an orange solid arrow indicate the X-ray path and the rotation direction of the entire system around the X-ray path, respectively. (*b*) Schematic illustration of the rotation discs. Wide slits are utilized for X-ray radiography observation, while narrow slits are used for 2D-XRD measurements. Orange solid arrows and a red square denote the rotation directions of the discs and the beam position, respectively. The ends of the narrow slits are machined into large-radius circles and filled with tungsten rods (indicated in blue).

The rotational slit system comprises two WC discs with an 8 mm thickness, each having four wide slits (2 mm) for X-ray radiography and four narrow slits (0.1, 0.15 or 0.2 mm) for 2D-XRD measurements (Fig. 16 ▸). Each wide slit spans approximately 35° in azimuth, while each narrow slit covers approximately 30°. The ends of the wide slits are arc-shaped to suppress crack propagation caused by centrifugal forces. In contrast, the ends of the narrow slits are machined into large-radius circles and filled with tungsten rods. These slit-bearing discs are directly attached to pulse motors. The wide and narrow slits are arranged symmetrically to minimize axial runout during high-speed rotation. Notably, these slit-bearing discs have been designed for ease of interchangeability (within the experimental hutch) using a straightforward bolt-mounting mechanism. This flexibility enables the installation of custom ring-slit discs with various sizes or even narrower apertures, ensuring that the system remains highly effective and suitable for high-speed time-resolved XRD measurements at the exact same position; this is the case even when applying ultra-high pressures or handling very small samples (< 0.5 mm).

**Figure 17:**
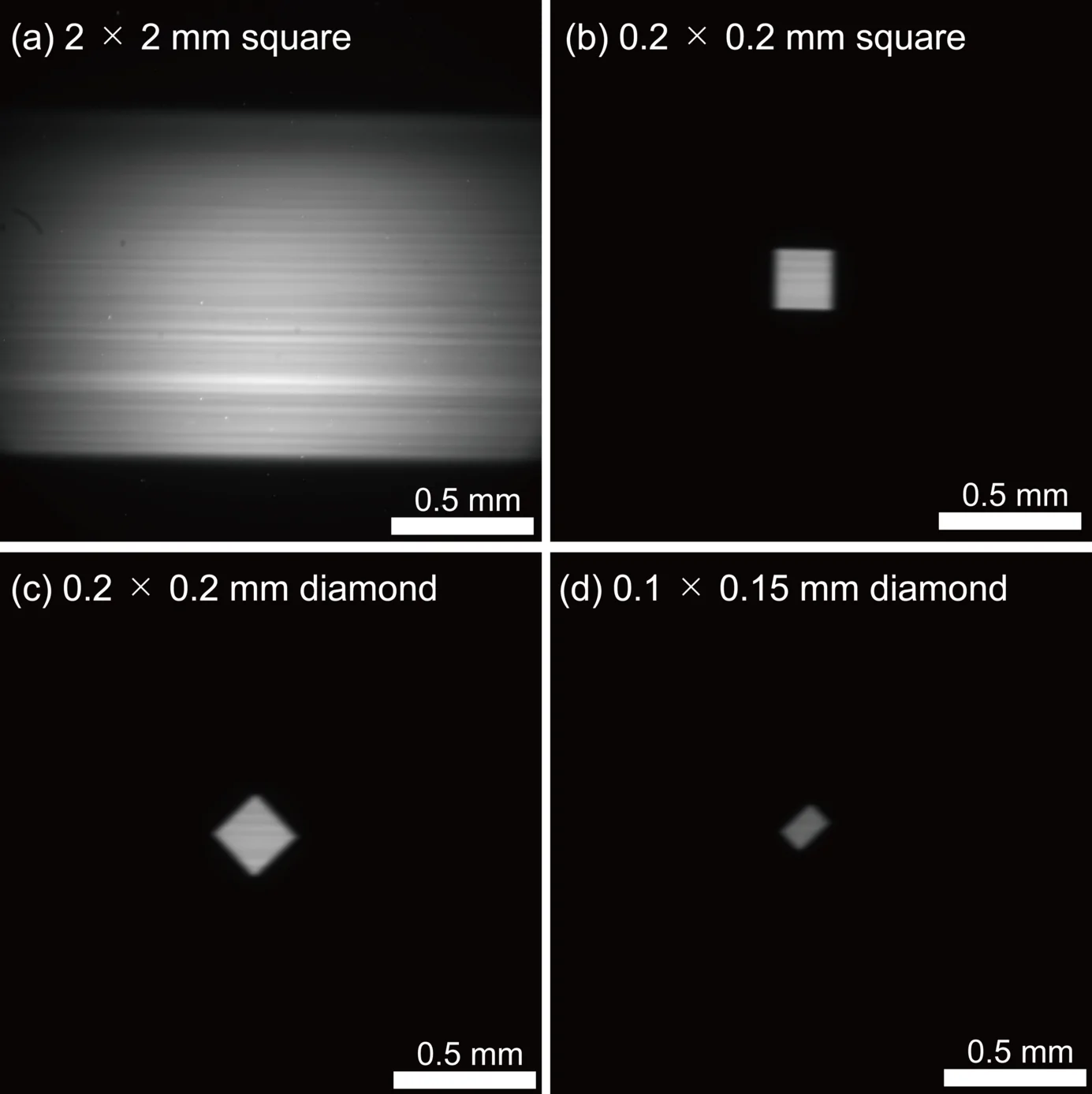
X-ray beam profiles observed through various slit configurations. (*a*) 2 mm × 2 mm wide slit configuration for X-ray radiography, (*b*–*d*) narrow slit configurations for 2D-XRD: (*b*) 0.2 mm × 0.2 mm square, (*c*) 0.2 mm × 0.2 mm diamond and (*d*) 0.1 mm × 0.15 mm diamond. The 2 mm × 2 mm wide slit configuration does not block any of the incident beam, of which full widths at half-maximum are approximately 1.2 mm vertically and 1.5 mm horizontally. The stripes in beam image (*a*) are caused by double-multilayer monochromators.

Synchronizing the rotation of the two discs enables rapid changes in slit size while maintaining the slit centre position [Figs. 17 ▸(*a*), 17 ▸(*b*)]. The positional repeatability of the four narrow slits for XRD is within 50 µm, accounting for the fixture wobble between the motor shaft and disc. The motorized φ*Y* and φ*Z* axes orient the rotation discs perpendicular to the X-ray beam, and the motorized *Y* and *Z* axes align the aperture with the beam centre. Additionally, the system can be manually rotated from −2° to 47° around the X-ray axis, which allows the XRD measurement area to be optimized for sample shapes both in uniaxial compression and shear experiments using a 45°-cut piston [Figs. 17 ▸(*c*), 17 ▸(*d*)].

The pulse motors rotating the discs operate at a rate of 0.72° per pulse, completing a full rotation in 500 pulses. The rotational speed of the discs reaches 2160 revolutions per minute (rpm) at the maximum pulse rate of 18000 pulses per second. Consequently, the radiography and XRD modes can switch at a maximum speed of 144 Hz. The minimum duration of X-rays via the narrow slits is approximately 2.7 ms. Each rotary motor has an absolute encoder counter with a 15-bit resolution (0.0109°), which can generate angle-specific trigger signals [5 V TTL (maximum 48 mA)] at an angular resolution of 0.1°. The XRD and radiography detectors acquire data thereby synchronized with alterations in slit aperture size. If necessary, attenuators can be installed solely over the wide slit gaps to protect the 2D-XRD detector from X-ray damage during radiography observation.

The key performance parameters and operational specifications of the endstations at the current SPring-8 facility are consolidated and summarized in Table 2 ▸.

## 3. Summary

BL15XU has been reconstructed as a high-energy X-ray beamline at SPring-8 to advance materials science and high-pressure research utilizing a 100 keV pink beam. The beamline provides ultra-brilliant undulator radiation quasi-monochromated by a double-multilayer monochromator, delivering a high flux of 6.0 × 10^13^ photons s^−1^ to experimental samples. In this article, we provide an overview of the beamline, covering its photon source, optics and experimental apparatuses. Additionally, the performance of each apparatus has been demonstrated through a scientific case study. EH1 focuses on materials engineering research using non-destructive observation techniques. The S3DXRD measurement system facilitates fatigue analysis of polycrystalline samples by visualizing constituent grains in terms of their position, orientation, shape and stress. The computed lamino­graphic imaging system is better suited for failure analysis of electrical parts or power devices implemented on a large flat circuit board. EH2 serves as a specialized hutch dedicated to high-pressure science, integrating the rotational slit system capable of rapid mode-switching between radiography and 2D-XRD at speeds up to 144 Hz. The installation of multiple large-volume presses facilitates comprehensive *in situ* observations under extreme conditions. These include the MADONNA press for investigations of deep-mantle phase transitions up to 120 GPa, the mobile Hyaku-shiki press for *in situ* deformation and acoustic emission measurements, and the Paris–Edinburgh press for PDF analysis of liquids and glasses. High-energy 100 keV X-rays further enhance PDF resolution by enabling data collection over a wide *Q* range up to 27.8 Å^−1^. Collectively, these integrated capabilities establish a versatile and powerful platform for fundamental and applied science across physics, chemistry and geoscience, ensuring the facility’s readiness for the future SPring-8-II upgrade.

## 4. Related literature

The following reference, not cited in the main body of the paper, has been cited in the supporting information: Kameshima *et al.* (2019 ▸).

## Supplementary Material

Supporting Information for article. DOI: 10.1107/S1600577526006387/tol5026sup1.pdf

## Figures and Tables

**Table 1:** Main source parameters of IVU-II22 generating 100 keV X-rays for SPring-8 and SPring-8-II

Parameter	SPring-8	SPring-8-II
Electron energy (GeV)	8	6
Storage current (mA)	100	200
Electron beam size (µm)	317.1 × 4.9	19.3 × 3.6
Electron beam divergence (µrad)	8.8 × 1.0	2.4 × 1.3
Undulator period (mm)	22	
No. of periods	149	
Undulator length (m)	3.278	
Magnetic pole gap (mm)	8.744	6.104
*K* value	1.36	1.97
Harmonic	7th	19th
Brilliance [photons s^−1^ mm^−2^ mrad^−2^ (0.1% bandwidth)^−1^]	2 × 10^19^	2 × 10^20^
Flux (photons s^−1^)†	1 × 10^14^	7 × 10^13^

† After the DMM described in the main text.

**Table 2:** Performance parameters and operational specifications of endstations

Category/station	Parameter/component	Specification/value
Beamline optics and source		
	Incident X-ray energy	100 keV
	Energy bandwidth (Δ*E*/*E*)	∼1% (pink beam)
	Photon flux at sample position	6.0 × 10^13^ photons s^−1^
	Maximum beam size (unfocused)	1100 µm vertical acceptance / ∼1.2 mm (V) × 1.5 mm (H) FWHM

EH1: materials engineering		
S3DXRD measurement system	Detector specification	Varex XRD 4343CT (resolution 2880 × 2880 / pixel size 150 µm × 150 µm)
	Sample-to-detector distance	1.45 m
	Maximum sample rotation speed	20° s^−1^
	Maximum *Q*	Up to 7.4 Å^−1^

Computed lamino­graphic imaging system	Detector specification	HB-25000-SB-M (resolution 5320 × 4600 / pixel size 2.74 µm × 2.74 µm)
	Sample-to-detector distance	100 mm
	Effective pixel size	0.274 µm × 0.274 µm (20× optical magnification and 2 × 2 binning)
	Effective field of view	712.4 µm × 630.2 µm (20× optical magnification and 2 × 2 binning)

EH2: high-pressure science		
Rotational slit system	Aperture configurations	2.0 mm wide slits (radiography) / 0.1, 0.15, 0.2 mm narrow slits (2D-XRD)
	Blade material / thickness	WC / 8 mm
	Maximum switching frequency	144 Hz
	Maximum X-ray exposure duration	∼2.7 ms (via narrow slits)

Large-volume press (MADONNA)	Guide block and press mechanism	D-DIA-type guide-block system/1500 ton uniaxial press (maximum load 15 MN / limited to 13 MN)
	Maximum generated pressure	Up to 120 GPa (utilizing sintered diamond anvils)
	2D-XRD detector type	Teledyne Rad-icon 2022 flat panel (99 µm element size, 220 mm × 220 mm)
	X-ray radiography imaging setup	Hamamatsu CMOS camera (C14440-20UP, 0.813 µm effective pixel size with 8× telecentric lens)
	Observable angular and *d*-spacing range	Full circle up to 2θ = 3.7° (1.92 Å); max. diffraction to 5.4° in 2θ (1.315 Å)

Mobile multi-anvil apparatus (Hyaku-shiki)	Press type and portability	Mobile DIA/D-DIA type (max. press load 200 tonf, total weight 5 ton)
	Pressure / temperature capabilities	Up to ∼20 GPa / pre-annealing test up to 1470 K
	Main supported measurements	*In situ* deformation experiments, AE monitoring
	2D-XRD detector type	WidePix 5 × 5 CdTe imaging detector

Paris–Edinburgh press setup	Standard cell assembly range	Up to 7 GPa and 2300 K (applicable for glasses and melts)
	CDT/DT cell assemblies range	Up to 12 GPa (cupped-Drickamer-toroidal)/17 GPa (double-toroidal) at room temperature
	Maximum accessible *Q* range	Up to 27.8 Å^−1^ (for PDF analysis of liquids and amorphous materials)
	Detector configuration	Dual point detectors: Amptek X-123 CdTe (low angles) and Mirion Technologies Ge (high angles)

## Data Availability

The data reported in the article are available from the corresponding authors upon reasonable request.
