# Supplementary material for: A high-energy X-ray beamline for materials science research at SPring-8: BL15XU
Source: J Synchrotron Radiat. 2026 Jul 27;33(Pt 5):1554–66. doi: 10.1107/S1600577526006387 (PMC13549348; doi:10.1107/S1600577526006387)
Supplement: Supplementary file 1 [file s-33-01554-sup1.pdf]

JOURNAL OF SYNCHROTRON RADIATION

Volume 33 (2026)

Supporting information for article:

A high-energy X-ray beamline for materials science research at SPring-8: BL15XU

Hiroyuki Ohsumi, Yuji Higo, Yujiro Hayashi, Jaemyung Kim, Noriyoshi Tsujino, Sho Kakizawa, Tetsuo Irifune, Tomohiro Ohuchi, Yoshio Kono, Hirokatsu Yumoto, Takahisa Koyama, Hiroshi Yamazaki, Yasunori Senba, Haruhiko Ohashi, Hidekazu Takano, Michihiro Sugahara, Taito Osaka and Makina Yabashi

Supporting Information for article:

A high-energy X-ray beamline for materials science research at Spring-8: BL15XU

Hiroyuki Ohsumi, Yuji Higo, Yujiro Hayashi, Jaemyung Kim, Noriyoshi Tsujino, Sho Kakizawa, Tetsuo Irifune, Tomohiro Ohuchi, Yoshio Kono, Hirokatsu Yumoto, Takahisa Koyama, Hiroshi Yamazaki, Yasunori Senba, Haruhiko Ohashi, Hidekazu Takano, Michihiro Sugahara, Taito Osaka and Makina Yabashi

1 Impact of beam tails on voxelized orientation map

In S3DXRD, the crystallographic orientation of each voxel is determined from the diffraction peaks assigned to that specific voxel. To identify the relevant diffraction images for a given voxel, the trajectory of the incident X-ray beam within the specimen is calculated for each rotation angle based on the measurement geometry, including the sample position and rotation angle. For every rotation angle, the X-ray beam traverses a distinct region in the specimen. When the selected voxel falls under the trajectory of the incident beam, the related diffraction image is employed for orientation determination. However, an actual X-ray beam profile includes low-intensity tails that can affect the real-space resolution of the reconstructed volume data. Exposure to regions outside the nominal trajectory of the X-ray beam within the specimen may cause crosstalk among neighboring voxels.

In this demonstration, the grains in the α-Fe wire specimen were larger than the nominal X-ray beam size. Consequently, the influence of low-intensity tails on the reconstructed grain morphology is not expected to be significant. However, for small or comparable grain sizes relative to the X-ray beam size, low-intensity tails may result in more pronounced distortion of the reconstructed volume data. In such cases, the possible influence of low-intensity tails should be carefully considered during data interpretation.

2 Beam profile after passing a four-quadrant slit

The horizontal and vertical beam profiles, together with Gaussian fits, are shown in Fig. S1(a) and (b), respectively. The zero positions for the horizontal and vertical slit blades, made of 5.0-mm-thick tantalum, were determined by measuring the complete beam blockage. The slit openings were subsequently set to 20 µm in both the horizontal and vertical directions. The resulting beam profiles were measured directly using an X-ray detector (Kameshima *et al.*, 2019), temporarily placed 950 mm downstream of the slit. The horizontal profile displays a Gaussian-like shape, with a nominal width of approximately 20 µm. In contrast, the vertical profile shows a shape closer to a top-hat intensity distribution.

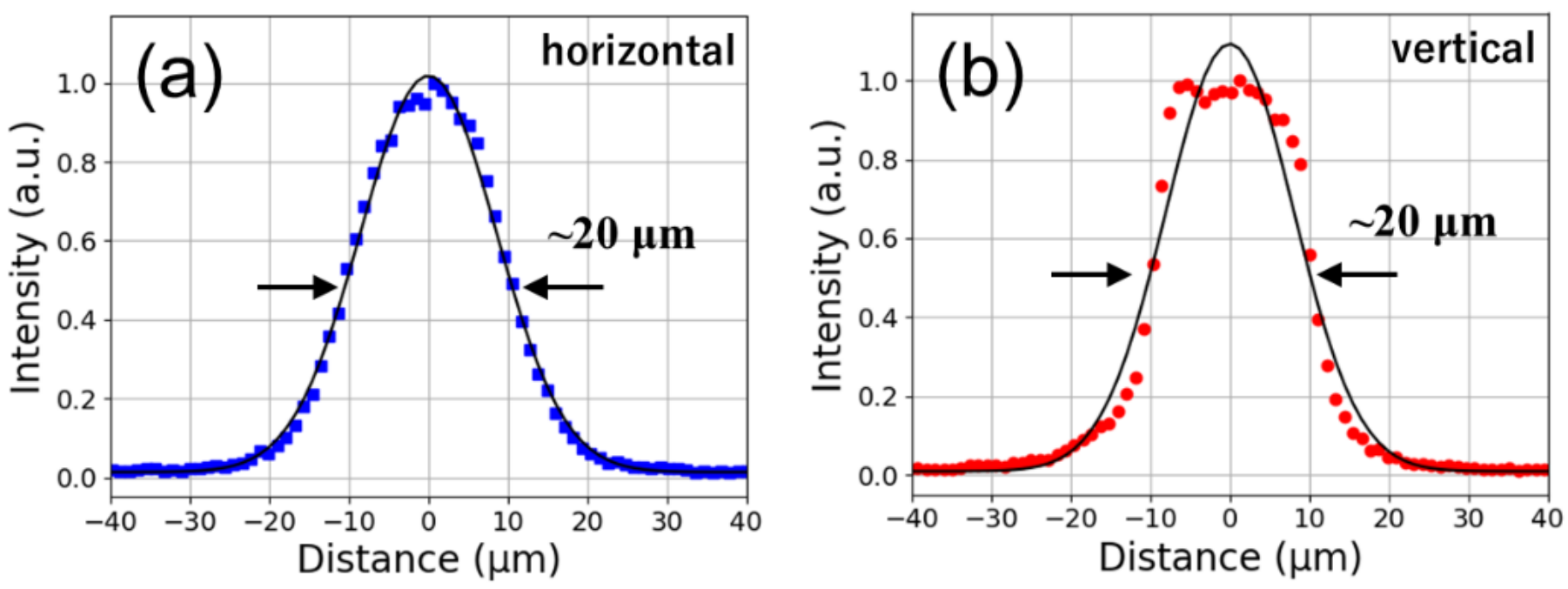

Figure S1 Measured beam profiles in the horizontal and vertical directions are shown in (a) and (b), respectively, together with Gaussian fits.

References

Kameshima, T., Takeuchi, A., Uesugi, K., Kudo, T., Kohmura, Y., Tamasaku, K., Muramatsu, K., Yanagitani, T., Yabashi, M. & Hatsui, T. (2019). *Opt. Lett.* **44**, 1403–1406.
